# Effectiveness of office-based vergence/accommodative therapy with movement for accommodative dysfunction in concussion-related convergence insufficiency: a secondary analysis of the CONCUSS randomised clinical trial

**DOI:** 10.1136/bjsports-2025-111200

**Published:** 2026-07-02

**Authors:** Mitchell Scheiman, Tara L Alvarez, Farzin Hajebrahimi, Melissa Noble, Suril Gohel, Rebecca Baro, Josie A Bachman, Christina Master, Arlene Goodman, CONCUSS Investigator Group, Katie Rownd

**Affiliations:** 1 Optometry, Drexel University, Philadelphia, Pennsylvania, USA; 2 Department of Biomedical Engineering, New Jersey Institute of Technology, Newark, New Jersey, USA; 3 Department of Health Informatics, Rutgers University School of Health Professions, Newark, New Jersey, USA; 4 Optometry, Drexel University, Elkins Park, Pennsylvania, USA; 5 Department of Pediatrics, The Children’s Hospital of Philadelphia, Philadelphia, Pennsylvania, USA; 6 University of Pennsylvania Perelman School of Medicine, Philadelphia, Pennsylvania, USA; 7 Somerset Pediatric Group, New Jersey Institute of Technology, Newark, New Jersey, USA

**Keywords:** Brain Concussion, Vision, Ocular, Exercise Therapy

## Abstract

**Objectives:**

To determine the effectiveness of office-based vergence/accommodative therapy with movement and home reinforcementt (OBVAM) for improving visual accommodative amplitude and facility in concussion-related convergence insufficiency with coexisting accommodative disorders.

**Methods:**

In this secondary analysis of the CONvergence Insufficiency in Persistent Post-CUSsion Symptoms (CONCUSS) randomised clinical trial, 79% (84/106) of participants with persisting symptoms 4–24 weeks after their last concussion, aged 11–25 years, had visual accommodative disorders. Participants were randomised to immediate OBVAM (12 sessions, two times per week) or delayed OBVAM for 6 weeks of watchful waiting. Then the outcome time 1 assessment compared accommodative amplitude (the maximum accommodative demand measured in dioptres (D) that a person can perceive clearly) for each eye monocularly and accommodative facility (a measure of how many times a participant could see an image clearly through a +2 D and −2 D lens within 1 min) between groups. Following outcome time 1 assessment, the immediate group completed four additional OBVAM sessions and the delayed group completed 16 OBVAM sessions two times per week. Outcome time 2 assessment occurred with both groups completing 16 OBVAM sessions. Analyses compared within and between group changes to assess the impact of therapy timing and additional sessions.

**Results:**

For outcome time 1 assessment, the immediate group (after 12 OBVAM sessions) showed significantly greater improvements (p<0.001) in accommodative amplitude, right eye: 4.7 D (95% CI 3.7 to 5.7) (6.3 cm); left eye: 5.1 D (95% CI 4.0 to 7.1) (6.8 cm), compared with the delayed group (watchful waiting), right eye: 1.0 D (95% CI 0.2 to 1.8) (1.3 cm); left eye: 1.0 D (95% CI 0.1 to 1.9) (1.3 cm). Accommodative facility at outcome time 1 assessment also improved more in the immediate group (5.1 cycles per minute (cpm) (95% CI 2.7 to 7.5)) than in the delayed group (2.4 cpm (95% CI 0.3 to 4.5)). For outcome time 1 assessment, participants with accommodative insufficiency, 86% in the immediate group, achieved resolution compared with 28% in the delayed group. No significant difference was observed between outcome time 1 and 2 assessments for the immediate group, comparing the 12–16 OBVAM sessions for accommodative function. When comparing the immediate and delayed groups at the outcome time 2 assessment (both groups received all 16 OBVAM sessions), no significant differences were observed between groups in accommodative function.

**Conclusion:**

OBVAM improves accommodative amplitude and facility following concussion. While delayed treatment did not impact the improvement of accommodative function, immediate therapy accelerates recovery and fosters a faster return to activities compared with delayed treatment. The CONCUSS study has previously reported an improvement in oculomotor convergence function, with a reduction in visual symptoms post-OBVAM compared with baseline measurements. If accommodative or convergence dysfunctions are identified in those with persisting symptoms after concussion, then intervention is recommended to expedite return to learn, sports and work.

**WHAT IS ALREADY KNOWN ON THIS TOPIC**

- Accommodative disorders are common after a concussion and have a high comorbidity with concussion-related convergence insufficiency. While vision rehabilitation is effective for accommodative disorders without head injury, the effectiveness of treatment for concussion-related accommodative disorders is unknown, given the additional symptoms of photophobia, dizziness and nausea experienced.

**WHAT THIS STUDY ADDS**

- The CONvergence Insufficiency in Persistent Post-CUSsion Symptoms (CONCUSS) randomised clinical trial evaluates the effectiveness of vision rehabilitation compared with watchful waiting, the impact of delayed treatment on therapeutic effectiveness, and the difference between 12 and 16 therapy sessions. Immediate treatment of 12 office-based vergence/accommodative therapy with movement and home reinforcement (OBVAM) sessions remediates 86% of accommodative dysfunction compared with only 28% in the watchful waiting group. While 12 OBVAM sessions remediate accommodative dysfunction in most patients, an additional 4 OBVAM sessions (16 total) may be helpful for some patients. When both groups completed 16 OBVAM sessions, significant differences were not observed. Immediate treatment is recommended because it fosters a faster return to activities.

**HOW THIS STUDY MIGHT AFFECT RESEARCH, PRACTICE OR POLICY**

- The CONCUSS study shows that immediate accommodative treatment is effective, and the accommodative system, on average, does not remediate with natural recovery when a patient is symptomatic at least 4 weeks postinjury. While 12 sessions are typically successful in remediation for most patients, some require an additional four sessions.

## Introduction

 Accommodative disorders are highly prevalent in children with typically-occurring convergence insufficiency (TYP-CI) unrelated to concussion.^1–5^ In the Convergence Insufficiency Treatment Trial (CITT)^1^ and CITT-Attention and Reading Trial (ART)^2^ Studies, 74% (164/221) and 69% (215/310) of participants with TYP-CI, respectively, exhibited coexisting accommodative disorders. These randomised clinical trials (RCTs) provide strong evidence that office-based vergence/accommodative therapy effectively enhances both accommodative amplitude and facility in children with TYP-CI.^1 2^ However, comparable data on the effectiveness of vergence/accommodative therapy for accommodative disorders associated with concussion-related convergence insufficiency (CONC-CI) are lacking. This gap in the literature is important because multiple studies have reported a high prevalence of CONC-CI and associated accommodative disorders.^6–10^ Reported prevalence rates of accommodative disorders in this population range from 35% to 84%.^6–9^

CONC-CI differs substantially from TYP-CI in both aetiology and clinical presentation. CONC-CI arises acutely following head injury and is frequently accompanied by a broader constellation of postconcussion symptoms, including photophobia, dizziness, nausea, cognitive fatigue, motion sensitivity and mood and sleep disturbances.^11 12^ These systemic symptoms can amplify visual discomfort and may influence both examination findings and treatment responsiveness. In contrast, the onset of TYP-CI is less clearly defined and not accompanied by such symptoms. Additionally, individuals with CONC-CI demonstrate a higher prevalence of co-existing accommodative deficits than those with TYP-CI, contributing to a more complex clinical profile.^6–10^ Because of these distinctions, treatment outcomes established in TYP-CI populations cannot be assumed to generalise directly to patients with CONC-CI, underscoring the need to evaluate the effectiveness of vergence and accommodative therapy specifically in a concussion population.

In the CONvergence Insufficiency in Persistent Post-CUSsion Symptoms (CONCUSS),^10 13^ 106 participants were randomised to immediate office-based vergence/accommodative therapy with movement (OBVAM) or delayed OBVAM treatment by 6 weeks. OBVAM therapy was a structured, therapist-guided programme designed to improve deficits in the vergence and accommodative systems.^13^ The ‘movement’ component refers to the integration of dynamic head and body positioning with traditional vergence and accommodation tasks to more closely simulate real-world visual demands. This combined approach aims to enhance visual function by engaging sensorimotor pathways that are often disrupted following concussion. A prior publication^10^ reported that 12 1-hour sessions of OBVAM improved near point of convergence (NPC) and positive fusional vergence (PFV) in 88% of patients with CONC-CI. In contrast, spontaneous recovery was uncommon, with only 8% of patients improving after 6 weeks of watchful waiting. Here, we evaluate the effectiveness of OBVAM in improving accommodative amplitude and facility in participants with CONC-CI and coexisting accommodative disorders.

## Participants and methods

The CONCUSS National Institutes of Health-funded RCT is registered at www.clinicaltrials.gov (NCT05262361). The full trial protocol can be accessed in an earlier publication.^10^

### Study design

The CONCUSS RCT study design was a parallel group with a 1:1 allocation ratio. Participants were randomly assigned to either immediate office-based vergence/accommodative therapy with movement and home reinforcement (OBVAM) or delayed OBVAM therapy (see figure 1). Complete details of the randomisation and allocation are available in previous papers.^10 13^

**Figure 1:**
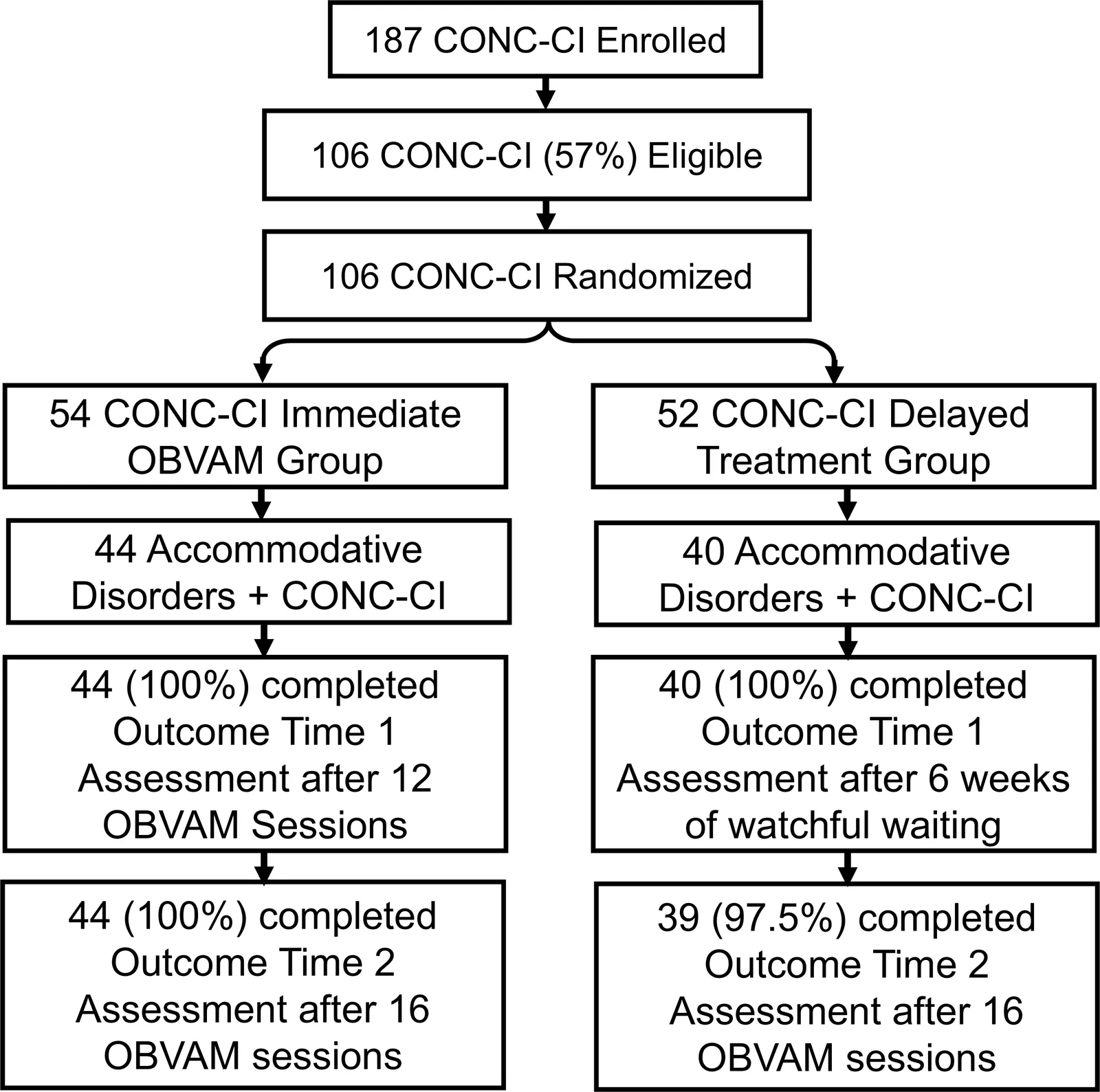
Flow chart of CONCUSS randomised clinical trial. CONC-CI, concussion-related convergence insufficiency; CONCUSS, CONvergence Insufficiency in Persistent Post-CUSsion Symptoms; OBVAM, office-based vergence/accommodative therapy with movement and home reinforcement.

### Participant selection

Participants were recruited from a private practice (coauthor AG) in New Jersey and the Minds Matter Concussion Program at the Children’s Hospital of Philadelphia (coauthor CM) between 16 November 2021 and 8 July 2024. Eligible participants, aged 11–25 years, had a medical diagnosis of concussion accompanied by persisting concussive symptoms, as determined by the referring physician, lasting 4–24 weeks postinjury. Persistent symptoms included ongoing postconcussion issues such as visual discomfort, dizziness, nausea or cognitive fatigue that had not resolved within the typical 4-week recovery. Enrolment criteria included symptomatic convergence insufficiency, characterised by: (1) an NPC that is receded (≥6 cm), (2) insufficient PFV, defined as failing Sheard’s criterion^14^ (base-out blur (break, if no blur) less than twice the near phoria) or having a minimum PFV at near of ≤15∆ base out break, and (3) a score of ≥16 on the Convergence Insufficiency Symptom Survey (CISS) for children aged 11–17 years or a score of ≥21 for adults aged 18 years and older.^15 16^ A study-certified masked examiner (coauthor MS) conducted a detailed sensorimotor vision assessment, which included subjective refraction, cover testing at distance and near, NPC evaluation, PFV at near, negative fusional vergence, near and distance vergence facility (12Δ base-out and 3Δ base-in), near random dot stereoacuity and accommodative testing, as outlined below. Although a single examiner conducted all assessments, masking was maintained through strict role separation. The examiner had no access to prior testing records, scheduling information or any indication of group assignment or visit sequence. All participant scheduling and interactions were handled by the research coordinator, who escorted each participant to the examiner without identifying the visit type. Because enrolment and visit timing occurred on a rolling, randomised basis, the likelihood that group assignment could be inferred at outcome assessments was reduced. This process ensured that the examiner remained masked throughout all assessments.

### Randomisation and masking

Participants were randomly assigned to either immediate office-based vergence/accommodative therapy with movement and home reinforcement (OBVAM), hereafter referred to as the immediate group, or delayed OBVAM therapy, hereafter referred to as the delayed group. Details of the randomisation procedure are available in previous publications.^10 13^ A study-certified examiner, masked to treatment allocation, conducted all baseline and outcome assessments.

### Measurement of accommodative function

We performed a non-cycloplegic refraction on all participants, adhering to established refractive criteria. All participants were required to meet these criteria on a routine basis. If a change in prescription was required, the participant had to wear the new prescription for 2 weeks before being re-evaluated. Since all participants were older children, adolescents and young adults, we opted to use a non-cycloplegic refraction method. Results indicated that there were very few children, with a mean age of ∼16 years old (SD ∼3 years). All accommodative and sensorimotor measurements were performed at the enrolment visit, before the initiation of OBVAM for the immediate group and before the watchful waiting period for the delayed group.

At baseline and during the two outcome visits, a masked examiner performed accommodation tests, including monocular accommodative amplitude (procedure shown here^17^) in each eye and monocular accommodative facility in the right eye. Accommodative amplitude is the maximum amount of accommodation available to meet an increasing accommodative demand, while accommodative facility is a 1 min test that assesses the endurance and response speed of the focusing system by repeatedly altering accommodative demand with alternating plus and minus lenses to mimic shifts between distance and near viewing. Participants wore their refractive correction, if needed, at all visits and for all examinations. Monocular accommodative amplitude was measured using the push-up method with a movable 20/30-sized vertical column of letters on the Astron Accommodative Rule (Bernell, Mishawaka, Indiana, USA, item GR50). The column of letters was gradually moved from a starting point of 25 cm toward the participant’s eye, as shown in our instructional video.^17^ The distance from the eye to the point where the participant first reported sustained blur of the letters was recorded to the nearest half-centimetre and then converted into dioptres (D) by dividing 100/distance in cm. For example, an accommodative amplitude of 20 cm is equivalent to 5 D (100/20), while a measurement of 5 cm is equivalent to 20 D (100/5). Thus, as the measurement in cm increases, the amplitude of accommodation in dioptres decreases, indicating a potential problem with accommodative amplitudes. Monocular accommodative facility was evaluated by counting the number of times a participant reported that a 20/30-sized vertical column of letters at 40 cm was clear when viewed through alternating +2.00 D and −2.00 D lenses. The number of flips per minute was counted and converted into cycles per minute (one cycle is viewing through both the positive and negative lenses). The expected finding for this test is 11 cycles/minute with a SD of 5 cycles/minute.^18 19^ Thus, a score of ≤6 cycles/minute is considered abnormal.

### Diagnostic criteria for accommodative disorders

Table 1 presents the criteria used to diagnose accommodative disorders in this cohort of participants experiencing symptomatic convergence insufficiency and persisting symptoms after concussion.

**Table 1:** Diagnostic criteria for accommodative disorders

Clinical diagnosis	Diagnostic criteria
Accommodative insufficiency	Must meet one of the following: Accommodative facility ≤6 cpm with difficulty clearing minus lens only. Amplitude ≥2 D below the age-expected norm (15 − 0.25×age (in years)).
Accommodative infacility	Must meet: Accommodative facility ≤6 cpm with difficulty clearing both plus and minus lenses.
Accommodative excess	Must meet both of the following: Accommodative facility ≤6 cpm with difficulty clearing plus lens only. Amplitude within or above the age-expected norm (≥15 − 0.25×age).
Accommodative dysfunction	Must meet both of the following: Accommodative facility ≤6 cpm with difficulty clearing plus lens only. Amplitude ≥2 D below the age-expected norm (15 − 0.25×age).

cpm, cycles per minute; D, dioptres.

### Treatment

Certified therapists followed a standardised protocol consisting of vergence, accommodative and movement-based therapy procedures.^20^ Therapists were recertified annually. These procedures were selected to systematically increase accommodative and vergence demands by varying lens power, viewing distance, target size, binocular versus monocular tasks and head–body movement. Typical accommodative therapy components included monocular and binocular accommodative amplitude activities, accommodative facility procedures using lenses and graded accommodative rock tasks. The OBVAM sequence followed pre-specified objectives and endpoints for each procedure, ensuring that participants progressed only after meeting measurable performance criteria (eg, sustaining clear focus, achieving target facility rates or consistently reaching specified vergence ranges). Descriptions of all procedures, movement elements and the corresponding objectives and endpoints that guided progression are provided in online supplemental table 1.

Both groups attended weekly 60-minute OBVAM visits for 16 sessions (two times per week), during which participants performed four to five therapy procedures under the guidance of a certified therapist. Additionally, therapy procedures were prescribed for 15 min per day, 3 days a week, to be performed at home on days that they were not participating in office-based therapy. Both groups also received standard concussion care from their physician. The type of standard concussion care was balanced between groups and reported previously.^10^

Although the primary purpose of the OBVAM was to treat convergence insufficiency, considerable effort was made to address accommodation, given the high frequency of comorbid accommodative disorders. The therapy programme included typical accommodative therapy procedures that used different lens powers, print sizes or viewing distances to change accommodative demand. These procedures were sequenced from monocular to binocular, based on the attainment of prespecified objectives and endpoints.^19 21^

### Outcome measures and assessments

The primary outcome measures were accommodative amplitude and facility. The immediate group completed 12 sessions of OBVAM over 6 weeks, while the delayed group only received standard concussion care during the first 6 weeks. Then, the outcome time 1 assessment was conducted for both groups. The outcome time 2 assessment took place after all 16 OBVAM therapy sessions were completed in both groups. This study design provided data to answer the following three clinical questions. The first clinical question was to determine whether 12 sessions of OBVAM are more effective than at least 6 additional weeks of watchful waiting (10–30 weeks following the most recent concussion) by comparing outcome time 1 assessments between the immediate and delayed groups. The second question was to evaluate treatment dosing by comparing 12 vs 16 OBVAM sessions in the immediate group, specifically by examining outcomes from the time 1 and 2 assessments. The third clinical question investigated the impact of at least an additional 6 weeks of watchful waiting (10–30 weeks after the most recent concussion) on treatment effectiveness by comparing outcome time 2 assessments between groups.

### Statistical methods

Data entry was conducted using the Research Electronic Data Capture (REDCap) portal at the Children’s Hospital of Philadelphia.^22^ Jamovi software, based on the R package, was used for statistical analyses. Baseline demographic statistics were calculated for each group using means and SD for continuous variables and frequencies for categorical variables. Unadjusted mean values (with 95% CIs) for the outcome variables were determined at baseline, outcome time 1 assessments and outcome time 2 assessments. To analyse the longitudinal repeated measures, a linear mixed-effects model^23^ was employed. The model included fixed effects for time (baseline, outcome time 1 assessment, outcome time 2 assessment), group allocation (immediate vs delayed) and their interaction (Time×Group), with random intercepts for participants to account for within-participant correlation. Covariates included age, sex, race, time since last concussion and number of prior concussions. All analyses used an intention-to-treat approach, incorporating all available data from baseline, outcome time 1 assessment and outcome time 2 assessment. Mean differences between baseline and each outcome time point were estimated from the Time×Group interaction. The 95% CIs were computed for all estimates. Post hoc Bonferroni corrections were applied to adjust for multiple comparisons when analysing within-group and between-group changes over time. Statistical significance was set at α=0.05. Cohen’s d effect sizes were calculated as the mean difference between outcome time 1 and outcome time 2 assessments, divided by the pooled SD of baseline scores. Effect sizes were interpreted as small (0.2<d < 0.5), moderate (0.5≤d <0.8) or large (d≥0.8).^24^

Furthermore, due to a well-established interaction between the vergence and accommodation systems through the accommodative convergence to accommodation (AC/A) and convergence accommodation to convergence (CA/C) crosslinks,^25 26^ the association between NPC and accommodative amplitude was examined using a linear mixed-effects model. Amplitude of accommodation was modelled as the dependent variable, with NPC included as a time-varying covariate, along with fixed effects for time, group and their interaction (while controlling for time since injury, number of concussions, sex, race and age), and a participant-specific random intercept to account for repeated measurements.

### Sample size and power considerations

This study was a secondary analysis of data collected as part of a previously published CONCUSS RCT,^10 13^ which included a priori sample size calculations for the primary clinical outcome measures. These calculations defined clinically meaningful differences and estimated sample sizes to ensure 80% power at α=0.05, ultimately leading to 50 participants per group. Therefore, no additional sample size calculations were performed for the present analysis. Importantly, a post hoc power analysis was not conducted, consistent with best practices and established statistical guidance.^27 28^ Instead, our statistical inferences are based on effect size estimates, CIs and model-based hypothesis testing using the linear mixed-effects model.

### Patient and public involvement in research

Feasibility studies used the sensorimotor examination to assess whether symptom severity in participants with recent concussion and persisting symptoms was tolerable during testing.^29 30^ Adjustments were made to ensure that most participants with concussion-related accommodative disorders could complete the assessments.

### Equity, diversity and inclusion statement

The study included six women and three men at various career stages, early, middle and late. Participants were eligible regardless of sex, gender, race/ethnicity or socio-economic background. The majority of participants were female (70%) and white (83%), mainly reflecting the usual demographics of each clinical site.

## Results

Between 16 November 2021 and 8 July 2024, 187 participants were evaluated; 106 CONC-CI participants were deemed eligible and enrolled at two clinical sites. Using the diagnostic criteria outlined in table 1, 79% (84/106) had one or more accommodative disorders. Of these, 69% (58/84) experienced accommodative insufficiency, 5% (4/84) had accommodative excess, 10% (8/84) had accommodative infacility and 23% (12/84) faced accommodative dysfunction. 4% (4/84) received multiple accommodative diagnoses. Table 2 shows that these diagnoses were evenly distributed across the two treatment groups.

**Table 2:** Baseline demographic and clinical characteristics by treatment group

	Immediate OBVAM n=44	Delayed OBVAM n=40
Sex	Female: 64%; Male: 36%	Female: 81%; Male: 19%
Age at baseline examination: mean years (SD)	16.9 (3.1)	16.2 (2.7)
Clinical findings, mean (SD)	Immediate OBVAM n=44	Delayed OBVAM n=40
Exodeviation at distance (∆)	1.3 (1.9)	0.8 (0.9)
Exodeviation at near (∆)	6.7 (4.8)	5.8 (3.5)
Near point of convergence break (cm)	12 (5)	11.2 (3.3)
Near point of convergence recovery (cm)	14.7 (5.5)	13.8 (3.6)
Negative fusional vergence blur/break (Δ)	9.6 (3.7)	10.5 (4.2)
Positive fusional vergence blur/break (Δ)	10.3 (3.2)	9.8 (3.2)
Near vergence facility, cpm (12∆ BO/ 3∆ BI)	8.4 (6.7)	9 (7.2)
Distance vergence facility, cpm (12∆ BO/ 3∆ BI)	2 (3.3)	3.5 (5)
Monocular accommodative amplitude, (D) OD	7.0 (2.2)	7.1 (2)
Monocular accommodative amplitude (D) OS	6.9 (2.2)	7.3 (2.2)
Monocular accommodative facility (cpm)±2.00 D	5.7 (5.9)	4.9 (5.6)
Clinical diagnosis	Immediate OBVAM	Delayed OBVAM
Accommodative insufficiency	66%	73%
Accommodative excess	11%	8%
Accommodative infacility	7%	3%
Accommodative dysfunction	23%	18%

OD is right eye and OS and left eye, D=dioptres, cpm=cycles/minute, ∆=prism dioptres.

OBVAM, office-based vergence/accommodative therapy with movement.

### Baseline characteristics

The baseline demographic and clinical measures are well-balanced between the immediate and delayed OBVAM groups (table 2).

To address the study’s three clinical questions: accommodative amplitude and facility were evaluated at baseline, after 12 OBVAM sessions for the immediate group (and 6 weeks of watchful waiting (clinical term for observation only and no formal treatment) for the delayed group) and again after both groups completed 16 sessions. This design allowed us to: (1) compare immediate treatment of 12 OBVAM sessions with watchful waiting at outcome time 1 assessment, (2) assess whether four additional sessions provided further benefit within the immediate group and (3) evaluate whether delaying treatment affected final outcomes by comparing groups at outcome time 2 when both groups completed 16 OBVAM sessions.

The within and between groups statistical tests are reported as differences between the three time points (baseline, outcome time 1 and outcome 2 assessments) for the three accommodative measurements (right eye and left eye amplitude of accommodation and accommodative facility) within table 3.

**Table 3:** Intention to treat analysis—differences in outcome measures at outcome time 1 and time 2 assessments, mean with (95% CI)

Clinical measures	Comparison		
	Baseline to outcome time 1		
Amplitude of accommodation right eye (dioptres) Mean (95% CI)	Immediate group	Delayed group	
	4.7 (3.7 to 5.7)	1.0 (0.2 to 1.8)	
	Baseline to outcome time 2		
	Immediate group	Delayed group	
	5.4 (4.3 to 6.6)	5.3 (3.9 to 6.7)	
	Outcome time 1 to outcome time 2		
	Immediate group	Delayed group	
	0.7 (0 to 1.4)	4.3 (3.3 to 5.4)	
	Between-group (outcome time 1)		
	IG vs DG	Significance P_Bonferroni_*	Effect size†
	3.5 (2.4 to 4.5)	**p<0.001**	1.6 (1.2 to 2.1)
	Between-group (outcome time 2)		
	IG vs DG	Significance p‡	Effect size†
	0.2 (−1.2 to 0.9)	p=0.77	0.03 (−0.4 to 0.5)
Amplitude of accommodation left eye (dioptres) Mean (95% CI)	Baseline to outcome time 1		
	Immediate group	Delayed group	
	5.1 (4.0 to 6.1)	1.0 (0.1 to 1.9)	
	Baseline to outcome time 2		
	Immediate group	Delayed group	
	5.9 (4.6 to 7.1)	5.3 (3.8 to 6.8)	
	Outcome time 1 to outcome time 2		
	Immediate group	Delayed group	
	0.8 (0.1 to 1.6)	4.3 (3.2 to 5.4)	
	Between-group (outcome time 1)		
	IG vs DG	Significance P_Bonferroni_*	Effect size†
	3.5 (2.4 to 4.6)	**< 0.001**	1.6 (1.1 to 2.1)
	Between-group (outcome time 2)		
	IG vs DG	Significance p‡	Effect size†
	0.1 (-1.1 to 1.2)	p=0.91	0.1 (-0.3 to 0.6)
Accommodative Facility (cpm), mean (95% CI)	Baseline to outcome time 1		
	Immediate group	Delayed group	
	5.1 (2.7 to 7.5)	2.4 (0.3 to 4.5)	
	Baseline to outcome time 2		
	Immediate group	Delayed group	
	7.8 (4.9 to 10.6)	9.9 (6.5 to 13.3)	
	Outcome time 1 to outcome time 2		
	Immediate group§	Delayed group§	
	2.6 (0.8 to 4.4)	7.5 (4.9 to 10.0)	
	Between-group (outcome time 1)		
	IG vs DG§	Significance P_Bonferroni_*	Effect size†
	3.9 (1.4 to 6.4)	**p=0.038**	0.7 (0.2 to 1.1)
	Between-group (outcome time 2)		
	IG vs DG§	Significance p‡	Effect size†
	1.0 (−1.6 to 3.6)	p=0.44	0.3 (−0.1 to 0.7)

* P Bonferroni corrected for multiple comparisons, bold font means statistically significant.

† Cohen’s D computed as the mean difference relative to the pooled SD of baseline scores.

‡ Unadjusted p value before correction for multiple comparisons.

§ Cycles per minute.

DG, delayed group; IG, immediate group.

### Effectiveness of 12 sessions of OBVAM therapy compared with watchful waiting for improving accommodative Function-Outcome time 1 assessment

The immediate and delayed between-group analysis comparing outcome time 1 assessment enabled us to compare the effectiveness of 12 sessions over 6 weeks of OBVAM to watchful waiting (table 3, figure 2).

**Figure 2:**
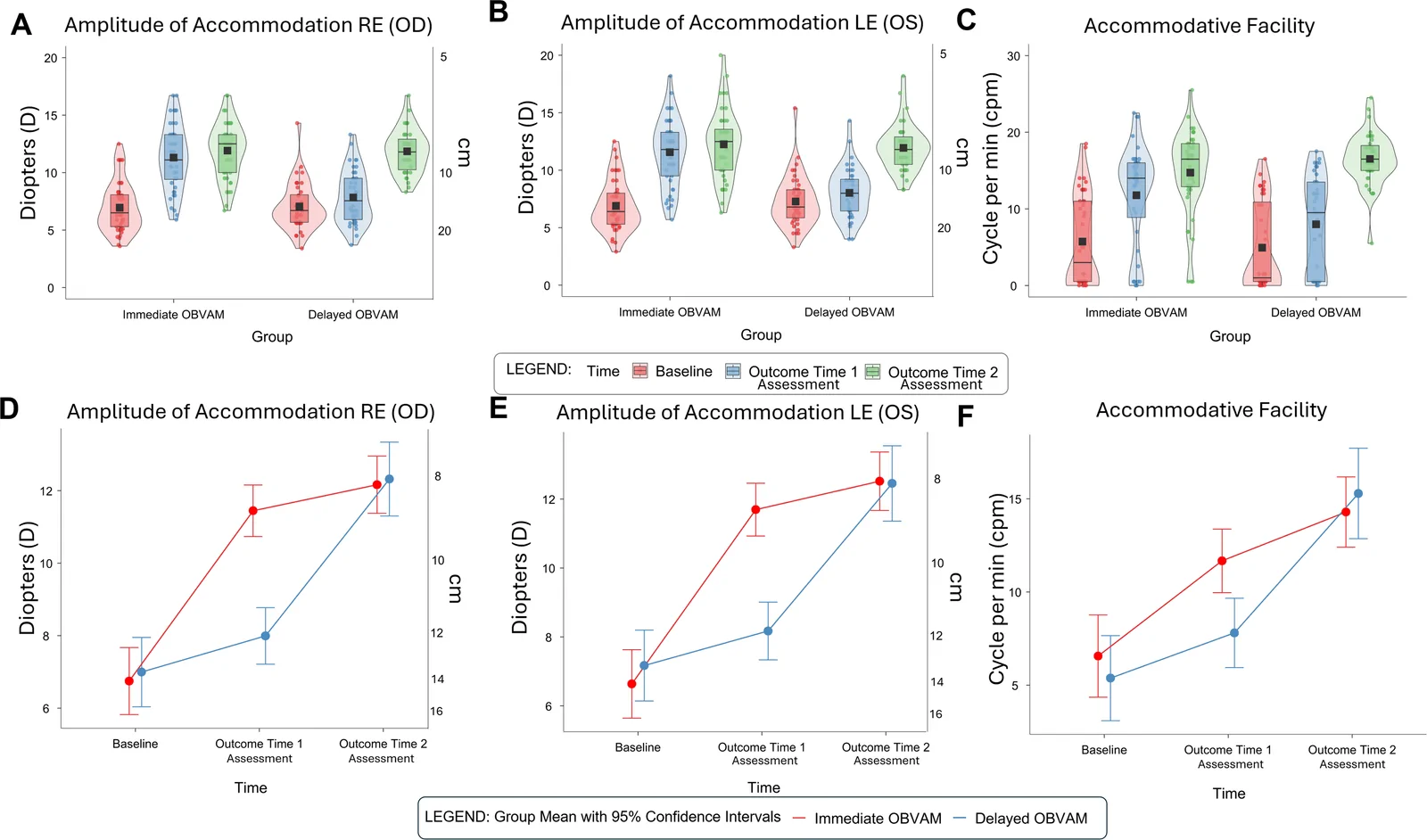
Clinical outcomes for accommodative function during the OBVAM intervention. (**A–C**) The distribution of the data using violin plots, showing the median (solid line), mean (square) and IQR (box) at each time point: baseline (red), outcome time 1 assessment (blue) and outcome time 2 assessment (green) for right eye accommodative amplitude (**A**), left eye accommodative amplitude (**B**) and accommodative facility (**C**). (**D–F**) Display the same underlying data, reorganised to emphasise between-group differences over time. Specifically, these panels present mean values with 95% CIs for the immediate and delayed groups across baseline, outcome time 1 and outcome time 2 for right eye accommodative amplitude (**D**), left eye accommodative amplitude (**E**) and accommodative facility (**F**). LE, left eye; OBVAM, office-based vergence/accommodative therapy with movement; OD, right eye; OS, left eye; RE, right eye.

*Accommodative Amplitude (right eye and left eye):* The immediate group demonstrated significantly greater improvement in accommodative amplitude than the delayed group in each of the left and right eye (p<0.001; Cohen’s d=1.6). The CIs indicate a statistically significant improvement in accommodative amplitude following immediate OBVAM.

*Accommodative facility (right eye*): The immediate group showed a higher mean accommodative facility than the delayed group at the outcome time 1 assessment (p<0.001). The difference between groups was also statistically significant (p=0.038) with a moderate effect size (Cohen’s d=0.7), and the CI indicates a notable improvement. Immediate therapy produced greater improvements than watchful waiting (table 3, figure 2).

### Per cent of participants achieving resolution of accommodative disorder

At baseline, accommodative disorders were common in both groups, with accommodative insufficiency identified in 66% of participants in the immediate group and 73% in the delayed group. Accommodative dysfunction was present in 23% and 18%, respectively. In contrast, accommodative excess and accommodative infacility were rare at baseline (immediate 11% and 7%; delayed 8% and 3%), and the small number of affected participants prevented meaningful condition-specific comparisons for these two diagnoses. Resolution was defined as no longer meeting the disorder-specific diagnostic criteria at follow-up (table 1). By outcome time 1 assessment, a higher proportion of baseline accommodative disorders had resolved in the immediate group compared with the delayed group for the two most common diagnoses, including accommodative insufficiency (86% resolved in the immediate group vs 28% in the delayed group) and accommodative dysfunction (90% resolved in the immediate group vs 14% in the delayed group). By outcome time 2 assessment, when both groups completed the 16 OBVAM sessions, resolution rates were high in both groups for these diagnoses, with accommodative insufficiency resolving in 93% of the immediate group and 97% of the delayed group and accommodative dysfunction resolving in 100% of participants in both groups, indicating improvement following 16 OBVAM sessions in both arms.

### Does a 6-Week delay affect the final results for the delayed group?—Outcome Time 2 Assessment?

*Accommodative amplitude (right and left eye*): At outcome time 2 assessment, the mean accommodative amplitude was not significantly different between the immediate and delayed groups for both the right eye (11.9 D (8.7 cm) versus 11.8 D (8.7 cm), respectively; unadjusted p=0.77, d=0.03) and the left eye (12.2 D (8.8 cm) vs 11.9 D (8.6 cm); unadjusted p=0.91, d=0.10). Note that for the results that were insignificant before correction for multiple comparisons, we have reported the unadjusted p values.

*Accommodative facility*: The delayed group had a slightly higher mean accommodative facility than the immediate group (16.5 cpm vs 14.7 cpm), but the difference was not statistically significant (unadjusted p=0.44). The effect size was small (Cohen’s d=0.3), and the CIs indicate minimal variability.

### The benefits of four additional therapy sessions in enhancing accommodative function

*Accommodative amplitude (right and left eye*): The mean accommodative amplitude improved slightly between the outcome time 1 assessment (12 OBVAM sessions) and outcome time 2 assessment (16 OBVAM sessions) in the immediate group for both the right eye (11.3 D vs 11.9 D, respectively) and the left eye (11.6 D vs 12.2 D, respectively). However, the differences were not statistically significant (p>0.5).

*Accommodative facility:* The mean accommodative facility improved slightly between the outcome time 1 and 2 assessments in the immediate group, 11.8 cpm versus 14.7, respectively. This change was not statistically significant (p=0.07) (table 3, figure 2).

#### Near point of convergence and accommodative amplitude association

The association between NPC and accommodative amplitude (figure 3) was evaluated using linear mixed-effects models that accounted for repeated measurements across time and participants. For both the left and right eye, NPC was a significant predictor of accommodative amplitude across all time points and groups. Specifically, worse NPC was associated with lower accommodative amplitude for the left eye (β = −0.32, SE=0.04, p<0.0001) and the right eye (β = −0.31, SE=0.04, p<0.0001), after adjusting for time, group, time since injury, number of concussions, sex, race and age. These findings indicate a robust, model-based association between vergence and accommodation measures that is consistent across repeated assessments. Importantly, the Time×Group interaction remained significant for both the left eye (p=0.0029) and the right eye (p=0.0023), demonstrating differential changes in accommodative amplitude over time between the immediate and delayed OBVAM groups, independent of the NPC–accommodation association.

**Figure 3:**
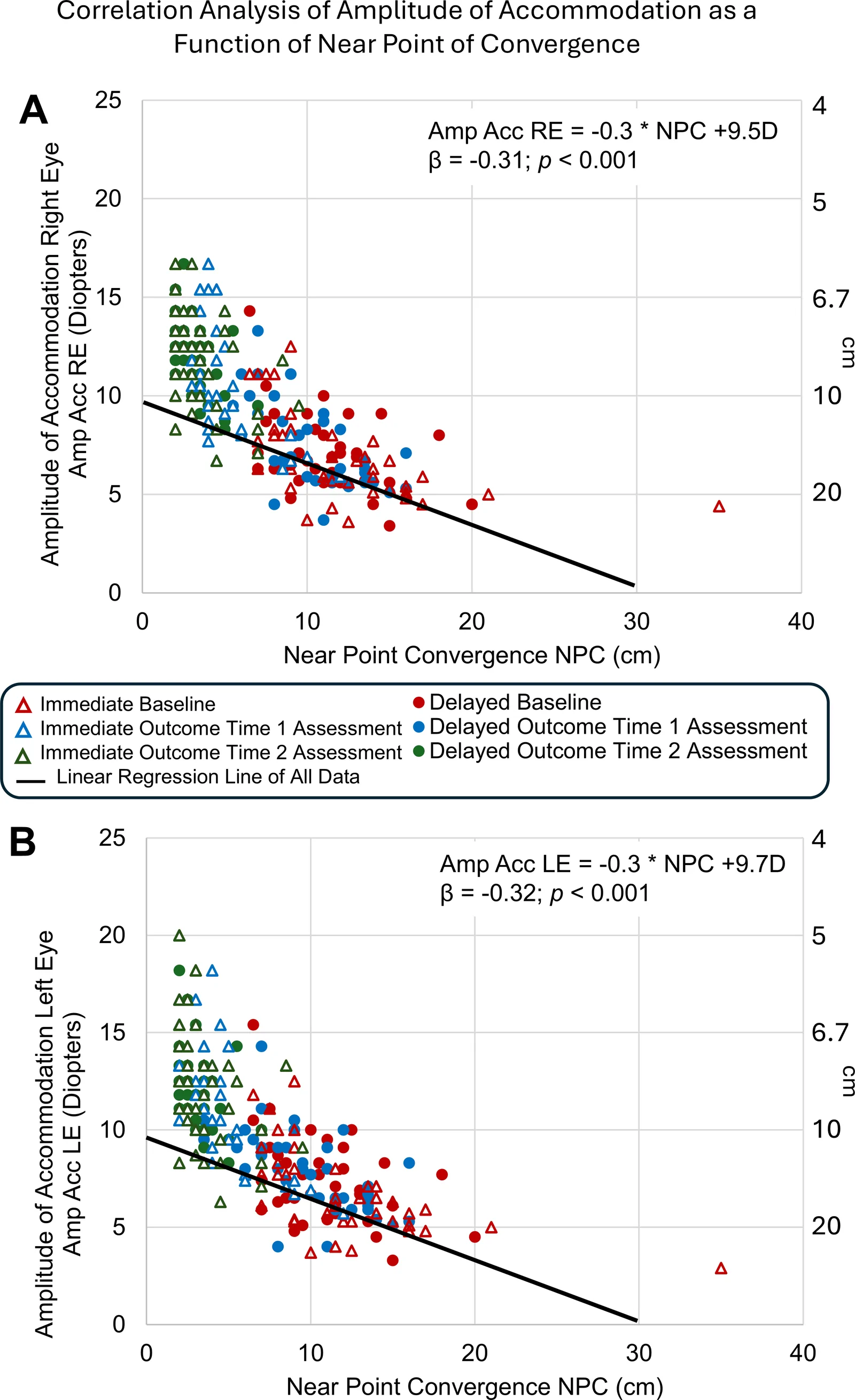
Association between the amplitude of accommodation and the NPC. Amplitude of accommodation for the right eye (**A**) and left eye (**B**) is plotted as a function of the near point of convergence (cm). Open triangles represent the immediate OBVAM therapy group, and solid circles represent the delayed OBVAM group. Symbol colours indicate assessment timepoints: red for baseline, blue for outcome time 1 assessment and green for outcome time 2 assessment. The solid black line represents the estimated fixed effect from the linear mixed model, which accounts for the nested nature of the data (three time points per participant). The model revealed a significant negative association for both the left eye (β=−0.318, p<0.0001) and the right eye (β=−0.309, p<0.0001), indicating that improvements in vergence (lower NPC) are significantly coupled with increases in accommodative amplitude. Amp Acc, amplitude of accommodation; LE, left eye; OBVAM, office-based vergence/accommodative therapy with movement; RE, right eye.

### Influence of clinical and demographic covariates

Analysis of clinical and demographic covariates—including time since injury, number of prior concussions, sex, race and age—demonstrated that these factors generally did not influence accommodative recovery. Two exceptions were noted for accommodative facility: age was a significant negative predictor (β = −0.42, SE=0.18, p=0.03), indicating that older participants showed relatively lower accommodative facility and number of prior concussions was a significant positive predictor (β = +0.94, SE=0.41, p=0.02), indicating that participants with more prior concussions showed relatively higher accommodative facility. Another study investigating participants 21–62 years of age also reports that accommodative facility is inversely correlated with age.^31^ These observed associations between age, prior concussions and accommodative facility should be interpreted cautiously, as these were secondary findings and may reflect underlying variability in recovery patterns rather than causal effects. All other covariates remained non-significant across measures (all p>0.17). Importantly, as these factors were included as covariates within the linear mixed-effects model framework, their influence was statistically controlled for, ensuring that the primary treatment effects reported for OBVAM remain independent of individual demographic or injury-related differences. Notably, time since injury was not a significant factor for the amplitude of accommodation in the left eye (p=0.3) or right eye (p=0.3), or accommodative facility (p=0.2), indicating that the timing of the intervention relative to the injury did not alter its effectiveness.

### Adverse effects

Two participants experienced concussions during the study, but neither was caused by OBVAM treatment. One was in the delayed group and sustained a concussion during an after-school activity after the fourth OBVAM session. The participant took a 1-week break but still completed the second assessment. The other was in the immediate group and had a concussion from a car accident after the 11th session. The participant took a 2-week break, received the 12th session and then had their first assessment. In total, only two participants (2 out of 84) had concussions, both of whom improved in their accommodative function, indicating these adverse effects did not influence the results. No other adverse effects occurred.

## Discussion

The results of this RCT provide strong evidence that OBVAM is an effective intervention for treating accommodative disorders in individuals with CONC-CI who continue to experience symptoms 4–24 weeks postinjury. After 12 OBVAM sessions, the immediate-treatment group demonstrated markedly greater improvement in accommodative amplitude (4.7 D right eye, 5.1 D left eye) and accommodative facility (5.1 cpm) than the delayed group (1 D both eyes and 2.4 cpm, respectively). These findings indicate that early intervention leads to more rapid improvement in accommodative function and do not support delaying therapy in hopes of spontaneous recovery. However, once both groups completed 16 OBVAM sessions, accommodative outcomes no longer differed significantly, demonstrating that a delayed start does not diminish the ultimate benefit of treatment. Clinically, this suggests that although logistical or financial barriers may delay therapy initiation, meaningful improvement remains achievable. Nonetheless, delaying treatment prolongs the period during which accommodative deficits and associated functional difficulties in school, work and sports remain unaddressed.

We also examined how many participants no longer met criteria for an accommodative disorder at outcome times 1 and 2. By outcome time 1 assessment, a larger proportion of baseline accommodative disorders had resolved in the immediate group compared with the delayed group for the two most common diagnoses, including accommodative insufficiency (86% resolved in the immediate group vs 28% in the delayed group) and accommodative dysfunction (90% resolved in the immediate group vs 14% in the delayed group). By outcome time 2 assessment, after both groups completed the 16 OBVAM sessions, resolution rates were high in both groups for these diagnoses, with accommodative insufficiency resolving in 93% of the immediate group and 97% of the delayed group, and accommodative dysfunction resolving in 100% of participants in both groups, indicating improvement after 16 OBVAM sessions in both groups. In the interest of brevity, we did not include an analysis of symptoms here because it is available in our prior manuscript.^10^ Previously, we reported a detailed analysis of participants’ symptoms using the CISS, showing that symptoms significantly improve when accommodative and vergence functions improve.

Results from three RCTs have demonstrated that office-based vergence/accommodative therapy is an effective treatment for accommodative dysfunctions associated with convergence insufficiency.^1 2 32^ However, all participants in these previous studies had TYP-CI and no history of concussion. The earliest of these trials, conducted by Cooper, Feldman, Selenow *et al*, evaluated five young adults (mean age 27 years) using an accommodative facility training protocol that included a true placebo control. They reported that accommodative amplitude improved from 5.25 D to 8.00 D following active treatment, whereas the placebo group showed no meaningful change. When the placebo group subsequently crossed over to receive active therapy, their accommodative amplitude increased from 5.10 D to 7.25 D. In the CITT study,^1^ accommodative amplitude improved by 9.9 D for the right eye and accommodative facility by 9.6 cpm after 12 therapy sessions, whereas in the CITT-ART study,^2^ accommodative amplitude improved by 8.6 D and accommodative facility by 12.6 cpm following 12 sessions. Thus, this is the first report indicating that OBVAM therapy is also an effective treatment for accommodative disorders in patients with persisting symptoms after concussion. This is necessary because there are differences in aetiology and presentation between CONC-CI and typically-occurring CI. First, the onset differs between the two: CONC-CI emerges acutely, whereas the onset of typically occurring CI is less clearly defined. In addition, CONC-CI has a more complicated presentation with a number of co-morbid conditions in CONC-CI, such as motion and light sensitivity, mood, as well as sleep changes.^11^ Third, previous studies primarily enrolled adolescents, whereas the current study enrolled both adolescents and young adults. This is important because accommodative amplitude naturally decreases (gets worse) with age,^33^ and it cannot be assumed that the effectiveness of this treatment would be similar in young adults as it is in adolescents. Finally, in the two previous studies, the control groups included a placebo/sham therapy group or other active therapies rather than an observation or no-treatment group.

Our study also examined another critical question regarding the effects of waiting for natural recovery. At outcome time 2 assessment, after both groups completed 16 therapy sessions, accommodative amplitude and facility no longer differed between the immediate and delayed groups, indicating comparable overall treatment effects. These findings show that OBVAM effectively improves accommodative function regardless of when therapy begins. However, delaying treatment prolongs the period during which patients continue to experience accommodative deficits that may interfere with return to school, work and sports.

The analysis of four additional therapy sessions in the immediate group revealed statistically insignificant and clinically modest improvements, with less than 1 D of change in accommodation amplitude in both the right and left eyes. Furthermore, the accommodative facility for the immediate group between outcome time 1 and 2 assessments was not statistically significant, with a modest clinical improvement of 2.6 cycles per minute. While most participants achieved normal accommodative function within 12 sessions, a subset benefited from extended therapy. These smaller changes in accommodative function suggest that additional therapy may benefit patients who have improved but have not yet achieved a successful outcome. We defined normal accommodative amplitude as ≥15–1/4 of the age in years. Using this criterion, 5% (2/44) of participants met the standard in either the right or left eye after four additional visits (16 OBVAM sessions total), but did not meet this criterion after 12 OBVAM sessions. This group of two participants had a mean accommodative amplitude of 5.7 D (right eye) and 5.6 D (left eye), which is worse than the overall group mean of 9.1 D (right eye) and 8 D (left eye), suggesting that those with worse amplitudes may require more therapy visits.

These results highlight the importance of continuing accommodative therapy beyond the initial phase, especially if clinical results and symptoms have not normalised after 12 sessions of OBVAM. This indicates that clinicians should monitor each patient’s progress and consider extending treatment for those who do not meet normal accommodative function criteria after the initial 12 OBVAM sessions. Balancing the goal of maximising treatment outcomes with minimising unnecessary therapy is vital for clinical efficiency and reducing patient burden in terms of time and cost. It is important to note that this study was not designed to determine the optimal number of therapy visits, but rather to demonstrate the effects of 12 and 16 visits for accommodation disorder associated with CONC-CI. Whether additional therapy beyond 16 visits would have led to further improvements remains unknown.

In a previous study using data from the CITT-ART RCT, the authors identified a relationship between accommodative amplitude and NPC results, noting that a co-existing accommodative disorder in symptomatic CI (not related to concussion) was associated with a more receded NPC.^2^ We observed similar findings in this study involving participants with CONC-CI and related accommodative disorders. The amplitude of accommodation in the right and left eyes is significantly correlated with the NPC. This observation aligns with our understanding of the inter-relationship between accommodation and vergence.^25 26^ This relationship is expected because the NPC task requires simultaneous activation of both the vergence and accommodative systems. As convergence increases during NPC testing, accommodative vergence is stimulated through the AC/A crosslink, while accommodation simultaneously elicits convergence via the CA/C linkage. In contrast, standard vergence testing attempts to isolate fusional vergence by keeping accommodative demand constant. Thus, when accommodative amplitude is reduced, the contribution of accommodative vergence to NPC is diminished, leading to a more receded NPC. This synergistic interaction helps explain why poorer accommodative function is associated with a worse NPC result.

In two previous papers,^8 34^ the authors identified a new accommodation diagnostic category and named it ‘accommodative dysfunction’. They noted that ‘the two most common accommodative anomalies following concussion are paradoxical; patients were unable to reach age-expected accommodative amplitudes, but were also unable to relax accommodation (ie, clear the plus lenses) on facility testing.’ This is atypical because, if a person has difficulty with accommodative amplitude, they are expected to have difficulty clearing the −2 D lenses during accommodative facility testing.^19^ Since no other accommodative diagnosis adequately describes this condition, a new condition termed accommodative dysfunction was introduced as a clinical diagnosis. We used this new category, along with its diagnostic criteria, in this paper and also found an unexpectedly high frequency of this condition (20%) in our sample. A potential explanation for this phenomenon is that the difficulty with plus lenses during accommodative facility testing may be due to the coexistence of convergence insufficiency and accommodative insufficiency. In an attempt to maintain binocular vision, a patient may overaccommodate to provide accommodative vergence, thereby supporting inadequate PFV.^19^ The continued use of excessive accommodation over time may cause a secondary accommodative excess, which would manifest as difficulty with the amplitude of accommodation, along with trouble regaining clear vision with +2 D lenses during accommodative facility testing.

Beyond the direct benefits observed in accommodative function, these findings emphasise the broader implications for care following a concussion. Since accommodative disorders are common after concussion and significantly impact daily activities such as reading, screen use, work and academic performance, physicians who manage patients with concussion should incorporate accommodative amplitude screening into their workup, along with the NPC. If such problems are present, OBVAM treatment could enhance the overall quality of life for this group. Overall, this study highlights the importance of OBVAM as a targeted approach for treating concussion-related accommodative issues. The evidence affirms its usefulness as an effective therapy with meaningful clinical implications for vision rehabilitation after concussion. Longer-term studies are necessary to evaluate the durability of treatment benefits and refine therapy protocols for personalised patient care. Additionally, future research could explore a clinical trial to see if accommodative therapy alone for patients with only accommodative dysfunction, without the fusional vergence component, is as effective as the OBVAM therapy used in this study. If so, this might reduce the number of therapy sessions needed.

The strengths of our study include an adequate sample size, randomisation of patients, a delayed treatment group and successful masking of the examiner. A limitation of the study is that it was not explicitly designed to investigate the effectiveness of therapy for accommodative function. However, because the treatment procedures that would be prescribed for participants with accommodation dysfunction alone would actually be more extensive, there is reason to believe that the results of this study may underestimate the potential for success. The results of the long-term study (with a 1-year follow-up) will be published in a separate manuscript. Another limitation is the use of non-cycloplegic refraction to determine refractive error. One could suggest that in children with active accommodative function, a cycloplegic refraction is indicated because uncorrected hyperopia could affect accommodative function. Since we recruited adolescents and young adults, we determined that a non-cycloplegic refraction was sufficient. Finally, a potential limitation is that, despite masking procedures, the examiner could have theoretically inferred group assignment at the outcome time 2 assessment based on differences in the interval between visits. However, because enrolment and follow-up occurred on a rolling basis and the examiner had no access to scheduling information, the likelihood of unmasking was minimised.

## Conclusion

Office-based vergence/accommodative therapy with movement is an effective treatment for improving accommodative amplitude and facility in children and young adults with accommodative disorders associated with concussion-related symptomatic convergence insufficiency. This study underscores the value of OBVAM as a targeted intervention for concussion-related accommodative disorders, with significant implications for concussion care for those with persisting symptoms after concussion. Within the context of this study, the OBVAM protocol led to measurable improvements in accommodative function and quality of life for individuals with persistent post-concussion symptoms. In addition, the results indicate that observation or watchful waiting alone does not appear to resolve the accommodative problem, suggesting that immediate therapy should be the treatment of choice. Overall, these data support the use of simple visual function screening tests by concussion physicians, rehabilitation professionals and paediatricians, with referral to optometrists or ophthalmologists with specialty expertise in concussion care for comprehensive assessment and targeted vision therapy when indicated.

## Supplementary material

10.1136/bjsports-2025-111200 online supplemental table 1

10.1136/bjsports-2025-111200 online supplemental file 1

## Data Availability

Data are available upon reasonable request.
