# Supplementary material for: Effectiveness of office-based vergence/accommodative therapy with movement for accommodative dysfunction in concussion-related convergence insufficiency: a secondary analysis of the CONCUSS randomised clinical trial
Source: Br J Sports Med. 2026 Jul 2;60(19):e111200. doi: 10.1136/bjsports-2025-111200 (PMC13453390; doi:10.1136/bjsports-2025-111200)
Supplement: Supplementary file 1 [file bjsports-60-19-s001.pdf]

Supplemental Table 1: Vergence/Accommodative/Movement Therapy Procedures (OBVAM). Initial when the participant has completed the task.

PHASE ONE (6 Visits)		
**Concussion-Related Saccadic Therapy**		**Date & Initial**
Level 1: 2 Pencils (still) – Horizontal (head still), slow to fast repeat Vertical repeat for Vergence (far and near)	no discomfort (1 min each eye movement type) 3 min total plus instruction	
Level 2: 2 Pencils (moving) – Horizontal (head still), slow to fast repeat Vertical and repeat Vergence (far and near)	no discomfort (1 min each eye movement type) 3 min total plus instruction	
Level 3: 2 Pencils (still & repeat moving) – Horizontal (head still), slow to fast walking forward and backward for saccade Horizontal/ Vertical and repeat Vergence	no discomfort	
Level 4: 2 Pencils – slow to fast walk in circle repeat activities of level 3	no discomfort	
Level 5: 2 Pencils – slow to fast rotating body (spinning top) repeat activities of level 3	Minimal discomfort (slight dizziness is ok)	
**Concussion-Related Pursuit Therapy**		**Date & Initial**
Level 1: Hanging Ball – left to right, body still	no discomfort	
Level 2: Hanging Ball – forward and backward, body still	no discomfort	
Level 3: Hanging Ball - body rocking left to right, opposite direction of ball movement Repeat for vergence Forward and Backward, the opposite direction of the ball	no discomfort	
Level 4: Hanging Ball – Circle around the swinging ball	no discomfort	
**Gross Convergence**	**Endpoint**	**Date & Initial**
Brock String (Level 1)	Convergence to a bead placed 2.5 cm from the nose	
Brock String (Level 2)	Voluntarily converge to 2.5 cm from the nose	
Brock String (Level 3)	Converge eyes with no string	
**Accommodation**	**Endpoint**	**Date & Initial**
Loose Lens Accommodation Rock (Level 1)	Clear +1.50/-3.00 D, 10 cycles per minute	
Letter Chart Accommodation Rock (Level 1)	Clear near chart at 33 cm, change fixation, and clear far Letter Chart at 3 m for 10 cycles per minute	
**Vergence Non-Computer**	**Endpoint**	**Date & Initial**
Vectograms (Quoits, Base-out/Base-in (3/1 ratio)	25 ∆ Base-out/12 base-in (letter L)<br>Every cm is 2.5 prism diopter	
**Vergence -Computer-Based**	**Endpoint**	**Date & Initial**
VTS4 (RDS) Base-out only	30 base-out with RDS large targets (blue)	
**PHASE TWO (4 Visits)**		
**Accommodation**	**Endpoint**	**Date & Initial**
Loose Lens Accommodation Rock (Level 2)	Clear +2.00/-6.00 D, 10 cycles per minute	
2 Letter Chart Accommodation Rock (Level 2)	Clear near chart at an age-appropriate distance and be able to clear to distance chart (difficulty is where the near card is placed and should be about 10 cm from the eyeball)	
**Vergence Non-Computer**	**Endpoint**	**Date & Initial**
Vectograms (Spirangle) Base-out/Base-in (3/1 ratio)	25 ∆ Base-out/12 base-in (letter L)	
Aperture Rule	30 ∆ Base-Out (Card 12), 15∆ Base-in (Card 6)	
Eccentric Circles	30 ∆ Base-out (12 cm)	
**Vergence -Computer-Based**	**Endpoint**	**Date & Initial**

VTS4 RDS Base-out/Base-in (3/1 ratio)	45Δ Based out with RDS large targets / 15 Base-in, repeat with small targets	
**PHASE THREE (3 Visits)**		
**Vergence Non-Computer**	**Endpoint**	**Date & Initial**
Vectograms (Quoits/Spirangle) Jump Vergence	25 Δ Base-out, 12Δ Base-in (letter L)<br>alternately fuse from top to bottom Vectogram	
Vectograms (Quoit Base-out) with head movement (left, right, up, down) with patient sitting (no more than 2 inch movement each way)	No discomfort, up to 10 base-out	
Eccentric Circles	30Δ Base-out (12 cm), 15Δ (6 cm) Base-in	
**Vergence -Computer-Based**	**Endpoint**	**Date & Initial**
VTS4 (RDS) Jump Vergence	Fuse 45Δ Base out to 12Δ Base-in for at least 15 repetitions in 1 minute in *Step jump* duction mode with small targets	
**PHASE Four (3 Visits)**		
**Accommodation**	**Endpoint**	**Date & Initial**
Aperture Rule / Flippers	Single, clear vision while viewing Card 6 through +2.00D and alternately -2.00D at least 13 cycles per minute without suppression	
**Vergence Non-Computer**	**Endpoint**	**Date & Initial**
Eccentric Circles Jump Vergence	• Jump from Base-out fusion (separation of 12 cm, 30Δ Base-out) to Base-In fusion (separation of 6 cm, 15Δ Base-in).<br>• Switch between Base-Out/Base-In fusion with the cards held 6 cm apart for 20 repetitions	
Vectograms walking forward and backward at 10 BO	No discomfort, up to 10 base-out (5 steps, 3 cycles Forward and Backward)	
Vectograms walking in a circle at 10 BO	No discomfort, up to 10 base-out	
**Vergence -Computer-Based**	**Endpoint**	**Date & Initial**
VTS4 (RDS) Jump Vergence	Fuse 45Δ Base-out to 15Δ Base-in for at least 15 repetitions in 1 minutes in *random jump* duction mode	
