# Supplementary material for: Effectiveness of office-based vergence/accommodative therapy with movement for accommodative dysfunction in concussion-related convergence insufficiency: a secondary analysis of the CONCUSS randomised clinical trial
Source: Br J Sports Med. 2026 Jul 2;60(19):e111200. doi: 10.1136/bjsports-2025-111200 (PMC13453390; doi:10.1136/bjsports-2025-111200)
Supplement: Supplementary file 2 [file bjsports-60-19-s002.pdf]

Title: **Functional Mechanism of Neural Control in Post-Concussion Convergence Insufficiency**

Short Title Convergence Insufficiency in Post-Concussive Symptoms

eIRB Number

Protocol Date:5/18/2021

Amendment 1 Date:	Amendment 3 Date:
Amendment 2 Date:	Amendment 4 Date:

Study Principal Investigator

Tara Alvarez
323 Martin Luther King Boulevard
Fenster Hall Room 611
Newark, NJ 07102
Phone 862-251-0148 (cell) 973-596-5272 (office)
email: alvarez@njit.edu

Site Principal Investigator

Christina L. Master
The Children's Hospital of Philadelphia
3401 Civic Center Blvd
Philadelphia, PA 19104
Phone: 215-590-1520
Email: masterc@email.chop.edu

TABLE OF CONTENTS

Table of Contents ... ii
Abbreviations and Definitions of Terms ... iv
Abstract ... vi
Protocol Synopsis ... vii
Table 1: Schedule of Study Procedures (Case subjects only) ... xii
Figure 1: Study Diagram (Case subjects only) ... xiii

1 BACKGROUND INFORMATION AND RATIONALE ... 1

1.1 INTRODUCTION ... 1
1.2 NAME AND DESCRIPTION OF INVESTIGATIONAL PRODUCT OR INTERVENTION ... 1
1.3 RELEVANT LITERATURE AND DATA ... 1
1.4 COMPLIANCE STATEMENT ... 3

2 STUDY OBJECTIVES ... 3

2.1 PRIMARY OBJECTIVE (OR AIM) ... 3
2.2 SECONDARY OBJECTIVES (OR AIM) ... 4

3 INVESTIGATIONAL PLAN ... 4

3.1 GENERAL SCHEMA OF STUDY DESIGN ... 4
3.1.1 Screening Phase and Initial Assessment ... 4
3.1.2 Study Treatment Phase ... 4
3.2 ALLOCATION TO TREATMENT GROUPS AND BLINDING ... 5
3.3 STUDY DURATION, ENROLLMENT AND NUMBER OF SITES ... 5
3.3.1 Duration of Subject Study Participation ... 5
3.3.2 Total Number of Study Sites/Total Number of Subjects Projected ... 5
3.4 STUDY POPULATION ... 5
3.4.1 Inclusion Criteria (examples) ... 5
3.4.2 Index/Case Subject Inclusion Criteria ... 5
3.4.3 Index/Case Subject Exclusion Criteria ... 6

4 STUDY PROCEDURES ... 7

4.1 SCREENING VISIT ... 7
4.2 STUDY TREATMENT PHASE ... 7
4.2.1 Initial Assessment ... 7
4.2.2 fMRI ... 7
4.2.3 Between Initial Assessment and Outcome Visit 1 (Arm 1) ... 8
4.2.4 Between Initial Assessment and Outcome Visit 1 (Arm 2) ... 8
4.2.5 Outcome Visit 1 ... 8
4.2.6 Between Outcome Visits (Arm 1) ... 8
4.2.7 Between Outcome Visits (Arm 2) ... 8
4.2.8 Outcome Visit 2 ... 8
4.3 SUBJECT COMPLETION/WITHDRAWAL ... 9
4.3.1 Early Termination Study Visit ... 9

5 STUDY EVALUATIONS AND MEASUREMENTS ... 10

5.1 SCREENING AND MONITORING EVALUATIONS AND MEASUREMENTS ... 10
5.1.1 Medical Record Review (case subjects) ... 10
5.1.2 Imaging ... 11
5.1.3 Other Evaluations, Measures ... 12
5.2 EFFICACY EVALUATIONS ... 13
5.2.1 Diagnostic Tests, Scales, Measures, etc. ... 13
5.3 SAFETY EVALUATION ... 14

6 STATISTICAL CONSIDERATIONS 15

6.1 PRIMARY ENDPOINT 15
6.2 SECONDARY ENDPOINTS 15
6.3 STATISTICAL METHODS 15
6.3.1 Baseline Data 15
6.3.2 Efficacy Analysis 15
6.3.3 Safety Analysis 15
6.4 SAMPLE SIZE AND POWER 15
6.5 INTERIM ANALYSIS 16

7 STUDY INTERVENTION 17

7.1 DESCRIPTION 17
7.1.1 Treatment Compliance and Adherence 17

8 SAFETY MANAGEMENT 18

8.1 CLINICAL ADVERSE EVENTS 18
8.2 ADVERSE EVENT REPORTING 18

9 STUDY ADMINISTRATION 19

9.1 TREATMENT ASSIGNMENT METHODS 19
9.1.1 Randomization 19
9.1.2 Blinding 19
9.1.3 Unblinding 19
9.2 DATA COLLECTION AND MANAGEMENT 19
9.3 CONFIDENTIALITY 21
9.4 REGULATORY AND ETHICAL CONSIDERATIONS 21
9.4.1 Data and Safety Monitoring Plan 21
9.4.2 Risk Assessment 22
9.4.3 Potential Benefits of Trial Participation 22
9.4.4 Risk-Benefit Assessment 23
9.5 RECRUITMENT STRATEGY 23
9.6 INFORMED CONSENT/ASSENT AND HIPAA AUTHORIZATION 23
9.6.1 Screening 24
9.6.2 Main Study 24
9.6.3 Consent/HIPAA Authorization Plan for Subjects Who Reach Age of Majority 24
9.6.4 Waiver of HIPAA Authorization 24
9.7 PAYMENT TO SUBJECTS/FAMILIES 25
9.7.1 Payments to subject for time, effort and inconvenience (i.e. compensation) 25

10 PUBLICATION 25

11 REFERENCES 25

ABBREVIATIONS AND DEFINITIONS OF TERMS

CI	Convergence Insufficiency
OBVAT	Office-based vergence/accommodative therapy
TYP-CI	Typically occurring convergence insufficiency
PPCS-CI	Persistent post-concussion symptoms with convergence insufficiency
SCC	Standard-community concussion care
PPCS	Persistent post-concussive symptoms
CISS	Convergence insufficiency symptom survey
CISS-Con	Convergence insufficiency symptom survey for Concussion
VET	Vergence Endurance Test
VisQuaL-T	Vision Quality of Life with Time Survey
VRS3	Vergence Ramp and Steps, Saccades, and Smooth Pursuit test
VBT	Vestibular and Balance Therapy

ABSTRACT

Context:

Adolescent and young adult concussion is considered a substantial health problem in the United States, with recent research showing that about half of patients with persistent post-concussion symptoms have convergence insufficiency. While studies have examined both the neural mechanistic change and effectiveness of office-based vergence/accommodative therapy (OBVAT) for typically occurring convergence insufficiency (TYP-CI), there is currently no validated treatment for PPCS-CI, nor is there research comparing the pathology of TYP-CI and PPCS-CI. Further research is needed to evaluate whether the diagnostic and management procedures effective for TYP-CI should be utilized for PPCS-CI. This randomized controlled trial seeks to compare the neural mechanistic differences between TYP-CI and PPCS-CI as well as to determine the underlying neural mechanism of OBVAT and its effectiveness when combined with SCC.

Objectives:

The primary objective of this study is to compare the effectiveness of SCC plus OBVAT compared to SCC only. The secondary objectives are to determine the underlying neural mechanism of OBVAT administered to patients with PPCS-CI as well as to compare the neural mechanistic differences between TYP-CI and PPCS-CI.

Study Design:

Randomized controlled trial.

Setting/Participants:

This study will include approximately 100 case subjects, ages 15 to 25 years, who present to the Children's Hospital of Philadelphia or to Somerset Pediatric Group for a concussion 1-3 months post injury who are visually symptomatic and have a diagnoses of convergence insufficiency. Data from an additional 50 sex and age-matched (within 5 years) subjects recruited from the local community as healthy controls has already been collected.

Study Interventions and Measures:

This study will compare the outcomes of adolescents and young adults with persistent post-concussive symptoms with convergence insufficiency who are randomized to standard-community concussion care (SCC) plus office-based vergence/accommodative therapy (OBVAT) versus those randomized to SCC alone to evaluate the effect of OBVAT in adolescents and young adults with PPCS-CI.

The primary endpoint of this study is the reduction in visual symptoms. This study will also assess the change in vision function measures and fMRI results of those that perform OBVAT compared to those who complete SCC. Additionally, this study will also assess the dose response of 12 OBVAT sessions as compared to 16 sessions.

PROTOCOL SYNOPSIS

**Study Title**	**Functional Mechanism of Neural Control in Post-Concussion Convergence Insufficiency**
**Funder**	National Eye Institute of the National Institutes of Health NEI R01EY023261 renewal pending (top 14%)
**Clinical Phase**	Phase III
**Study Rationale**	There is currently no validated treatment for PPCS-CI nor is there research comparing the pathology of TYP-CI and PPCS-CI. Further research is needed to evaluate whether the diagnostic and management procedures effective for TYP-CI should be utilized for PPCS-CI.
**Study Objective(s)**	**Primary**<br>• To compare the effectiveness of SCC plus OBVAT compared to SCC only.<br>**Secondary**<br>• To determine the neural mechanism of OBVAT for PPCS-CI compared to SCC<br>• To determine the effectiveness of 12 one-hour sessions compared to 16 one-hour sessions of OBVAT.<br>• To compare the differences between PPCS-CI and TYP-CI.
**Test Article(s)** *(If Applicable)*	Office-based vergence/accommodative therapy plus standard-community concussion care vs. standard-community concussion care alone.
**Study Design**	Randomized controlled trial.
**Subject Population key criteria for Inclusion and Exclusion:**	**Inclusion Criteria (case subjects)**<br>1. Males or females ages 15 – 25<br>2. Best-corrected visual acuity of 20/25 in both eyes at distance and near.<br>3. Willingness to wear glasses or contacts to correct refractive error, if needed.<br>4. Stereopsis of 500 sec arc using Randot Stereo Test.<br>5. Symptomatic PPCS-CI<br>6. Parental/guardian permission (informed consent) and if appropriate, child assent.<br>7. Diagnosis of convergence insufficiency defined as a receded near point of convergence of more than 6 cm, insufficient positive fusional vergence defined as less than 15 prism diopters (or positive fusional vergence less than twice the

	near phoria), and a near dissociated phoria of 4 prism diopters more exophoria compared to the far dissociated phoria<br><br>**Exclusion Criteria (case subjects)**<br>1. Previous off- or home-based vision therapy, orthoptics, home-based near-target push-ups, or pencil push-ups.<br>2. Amblyopia or constant strabismus or strabismus surgery.<br>3. High refractive error: Myopia ≥ 6.0D sphere; Hyperopia ≥ 5.0D sphere; Astigmatism ≥4.0D; Anisometropia >1.5D difference between eyes; prior refractive surgery.<br>4. Manifest or latent nystagmus evident clinically.<br>5. Systemic diseases that affect accommodation, vergence or ocular motility (i.e. multiple sclerosis, Graves' thyroid disease, myasthenia gravis, diabetes, chemotherapy, or Parkinson's disease).<br>6. Any medication known to affect accommodation or vergence.<br>7. Near point of accommodation >20cm<br>8. Vertical heterophoria greater than 1Δ.<br>9. Any eye care professional or multiple people from the same household participating in the study.<br>10. Non-removable metal in the body.<br>11. Subjects who are pregnant, planning on becoming pregnant during the study duration, or breastfeeding.<br>12. Subjects who are metal workers.<br>13. Parents/guardians or subjects who, in the opinion of the Investigator, may be non-compliant with study schedules or procedures.
**Number Of Subjects**	Approximately 150 subjects will be tested for eligibility in the study to ensure approximately 100 evaluable subjects complete the longitudinal study.
**Study Duration**	Each subject's participation will last until the completion of their OBVAT sessions. For arm 1 cases, their participation will last 11 weeks +/- 3 weeks. For arm 2 cases, their participation will last 17 weeks +/- 3 weeks.

**Study Phases**<br>Screening<br>Study Treatment<br>Follow-Up	**Screening and Initial Assessment**: All patients who present to the Children's Hospital of Philadelphia or Somerset Pediatric Group with a concussion date of injury 1-3 months ago will be screened for enrollment as case subjects using the protocol inclusion and exclusion criteria. Clinicians specializing in concussion management will identify potential subjects under their care in clinics after a standard clinical evaluation that consists of a detailed history and examination. A member of the study team will introduce the study to subjects deemed eligible to participate, obtain verbal consent to screening, and screen them against study inclusion and exclusion criteria. Eligible subjects will have time to ask questions and consider participation prior to consenting. Parental/guardian permission (informed consent) and, if applicable, child assent, will be obtained prior to any study related procedures being performed. After consenting to participate, subjects will complete an initial optometric exam, vision assessments, fMRI, and a patient-reported symptom questionnaire. Data from control subjects recruited from local colleges and universities who did not have a recent concussion have already been collected.<br><br>**Intervention**: Case subjects will be randomly assigned into one of two arms. Arm 1 will begin OBVAT immediately, while arm 2 will start with 6 weeks of SCC followed by the OBVAT intervention. A member of the study team will connect subjects with the study vision therapist who will perform their OBVAT sessions. Arm 1 subjects will complete 12 1-hour bi-weekly OBVAT sessions with the vision therapist in addition to SCC while arm 2 subjects complete SCC alone. After 6 weeks, subjects will complete outcome visit 1. After outcome visit 1, arm 1 cases will complete 4 more OBVAT sessions and then complete outcome visit 2. After the initial 6 weeks, arm 2 cases will complete 16 1-hour bi-weekly OBVAT sessions and then complete outcome visit 2. All case subjects will complete at-home reinforcement activities during their OBVAT intervention.
**Efficacy Evaluations**	Change in symptoms over the course of the intervention.<br>Rate of symptom remediation in OBVAT+SCC group compared to SCC alone.<br>Change in vision function and fMRI measures.

**Safety Evaluations**	A trained and annually certified vision therapist will monitor all subjects during their OBVAT sessions to ensure that the sessions are being performed safely. Patient-reported symptoms will be monitored during each OBVAT session. Dr. Mitchell Scheiman has over 45 years experience with OBVAT and will be training and certifying vision therapists. He will be available for any questions. In prior large scale phase 3 randomized clinical trials studying OBVAT on neurologically normal children and young adults under Dr. Scheiman's leadership, no adverse effects were reported.
**Statistical And Analytic Plan**	A Wilcoxon signed test will determine whether significant changes occur between the true mean measurement after OBVAT+SCC or SCC only. A paired *t*-test will also be used due to its robustness property as a justifiable comparison. The direct treatment effects due to interactions between groups and time periods (before and after therapy) will be analyzed with post-hoc tests such as Tukey's method. Adjustments for multiple comparisons will be done to ensure an error rate α-level of 0.05.
**DATA AND SAFETY MONITORING PLAN**	The PI will assure that the privacy of subjects, including identity and medical information, will be maintained at all times. In addition, the PI and Mitchell Scheiman, O.D., Ph.D. will oversee the study team and will actively monitor the study to ensure that it is carried out safely and that a subjects' symptoms do not increase sharply or over an extended period of time as a result of participation. A physician in the concussion clinic will examine any case subject that appears to show a regular increase in symptoms over the course of the study.

TABLE 1: SCHEDULE OF STUDY PROCEDURES (CASE SUBJECTS ONLY)

**Study Phase**	**Screening/Initial Assessment**		**Treatment/Intervention**	
**Visit Number**	**1**	**2**	**3**	**4**
**Study Days**				
Informed Consent/Assent	X			
Review Inclusion/Exclusion Criteria	X			
Medical Record Review	X		X	X
Optometric Examination	X		X	X
CISS-Con and VisQuaL-T	X		X	X
VRS3	X		X	X
VET	X		X	X
fMRI		X	X	X*
Pregnancy test**		X	X	X
Randomization		X		
Adverse Event Assessment				

*=Arm 2 only

**=Female subjects only

FIGURE 1: STUDY DIAGRAM (CASE SUBJECTS ONLY)

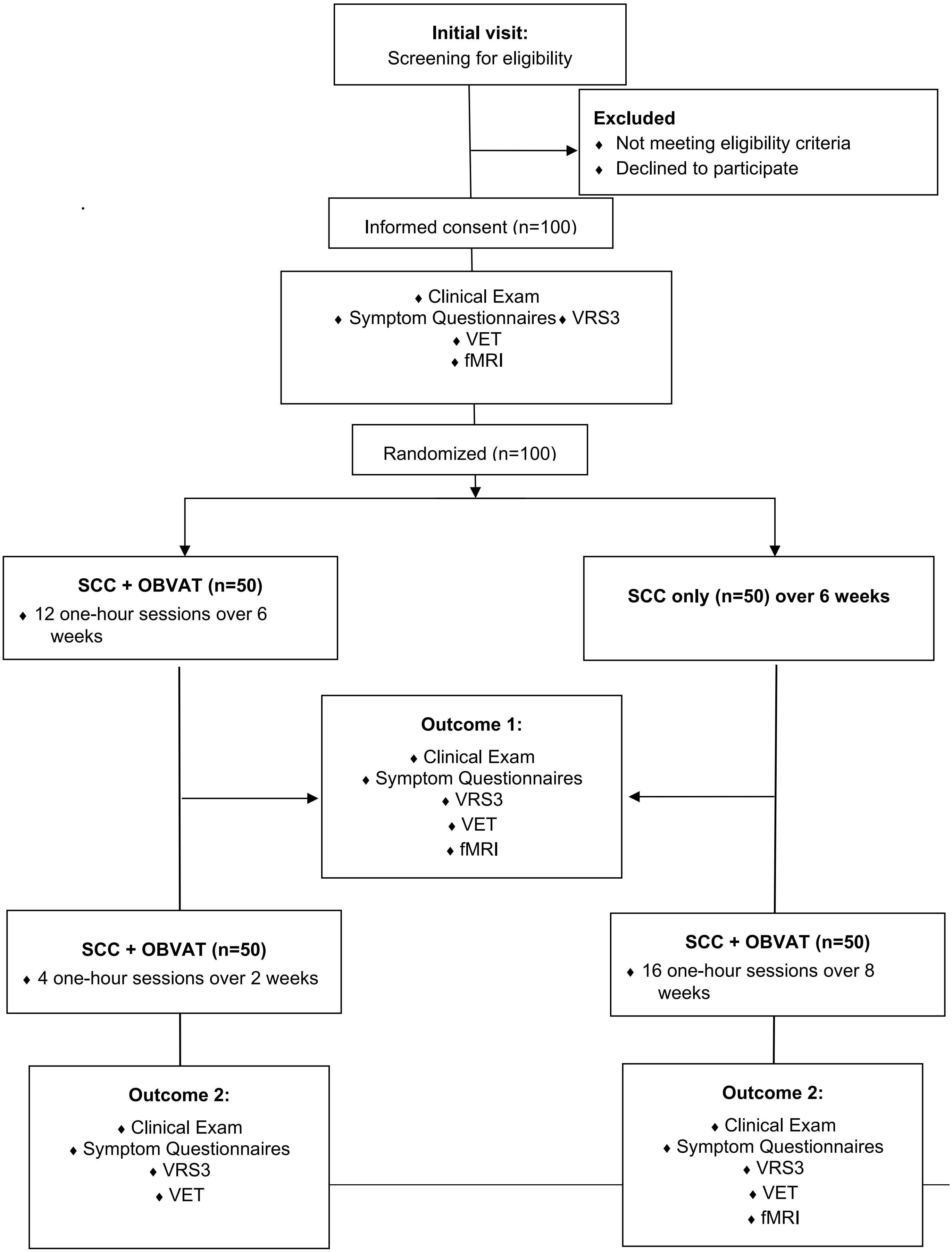

1 BACKGROUND INFORMATION AND RATIONALE

1.1 Introduction

Typically occurring convergence insufficiency (TYP-CI) is the most common binocular vision disorder in children and young adults. As such, recent literature has focused on the neural-mechanistic basis of, and treatment for, TYP-CI. These recent studies have been effective in remediating symptoms and improving vision in both children and adults using office-based vergence/accommodative therapy (OBVAT). However, these studies have not had participants with a history of head injury. The prevalence of convergence insufficiency (CI) in child and adults with persistent post-concussive symptoms (PPCS-CI) is higher than in the non-concussed populations, however there is no validated treatment for PPCS-CI

While the use of OBVAT in TYP-CI has been shown to be effective, its use in PPCS-CI has not been examined. This randomized controlled trial seeks to determine if OBVAT in addition to standard-community concussion care (SCC) improves recovery among adolescents and young adults with PPCS-CI (15 to 25 years of age).

1.2 Name and Description of Investigational Product or Intervention

This study involves an OBVAT intervention to determine if SCC + OBVAT improves convergence insufficiency for adolescents and young adults after concussion.

1.3 Relevant Literature and Data

Adolescent and young adult concussion is now considered a substantial health problem in the United States.[1–3] According to studies from BlueCross BlueShield between 2010 through 2015, the prevalence of concussion diagnosis has increased by 71% in the 10 to 19 year old age group.[4] While some symptoms resolve with supportive care, a longitudinal study showed that 41% of the 591 concussed patients studied were classified as having persistent post-concussion symptoms (PPCS) where symptoms did not dissipate after two weeks.[5] Another study reported that 46% of the 2946 enrolled children had a diagnosis of PPCS two-weeks post-injury which decreased to 33% four weeks post-injury.[6]

This study is important because PPCS has a profound impact on a person's ability to function scholastically [7-11] and professionally.[12-15] Adolescents are particularly vulnerable to the cognitive and developmental concussion consequences, often having a prolonged recovery and poorer outcomes than adults.[11,16] Oculomotor dysfunction is so common in PPCS that it is considered one of the phenotypes of concussion, owing to its frequent occurence.[17-21] Symptoms include dizziness, headaches, inability to sustain attention during a long duration task, fatiguing faster post-injury compared to pre-injury, difficulty with attention and memory, and blurry /double vision.[1,22-24] Convergence insufficiency (CI), is associated with many of the aforementioned characteristic symptoms.[25-28] In one study of PPCS, 70.4% of 260 concussed athletes report visual disturbances, which was the 7th most frequently reported symptom.[29]

CI is the most common non-strabismic binocular vision disorder in children and young adults, with prevalence estimates from school-based populations ranging from 3.4% to 12.7% for typically-occurring CI (TYP-CI) that is not associated with concussion.[30-36] Much higher rates of CI have been reported in children and young adults [26,37] with PPCS-CI, with estimates of 38% to 49% for children[38,39] and 44% to 47% for adults.[26,40] Since PPCS-CI is associated with a traumatic acceleration of the head resulting in injury, this suggests a different etiology than TYP-CI, which presumably develops over time and is not associated with head injury. In addition, vestibular dysfunction is commonly associated with PPCS-CI,[25,41,42] which is not the case for TYP-CI.[43] While both conditions share some similar clinical characteristics (receded near point of convergence, inadequate positive fusional vergence), the higher prevalence, sudden onset, and frequent comorbidity of vestibular dysfunction[44] combined with our pilot data suggest that PPCS-CI may be a unique physiological condition compared to TYP-CI. Objective eye movement recording with novel eye movement stimulus protocols to understand the underlying neural substrates assessed using functional MRI (fMRI) is needed. These measures will delineate the differences between the two CI subtypes since conventional and often subjective clinical measurements are not substantially different. With this knowledge, physicians will be positioned to develop more effective diagnostic and therapeutic strategies for the treatment of PPCS-CI, that can lead to faster return to school, sports, or work.

For TYP-CI, diagnostic protocols and therapeutic interventions are well-established[43,45-48] and results from multiple randomized clinical trials (RCT) summarized in a systematic Cochrane Review[49] show that office-based vergence and accommodative therapy (OBVAT) is an effective treatment in TYP-CI children[50-52] and young adults,[45,53,54] In fact, the PI along with Drs. Scheiman, Yaramothu, and Gohel are the only group to use objective eye movement recording with fMRI to show the underlying neural mechanistic differences between TYP-CI and binocularly normal controls (BNC) as well as the neural mechanism of how OBVAT changes the neurophysiology and visual function in TYP-CI. Our group and others have published pilot studies studying only a single treatment arm[40,55,56] and suggest that the same 12 one-hour sessions of OBVAT that is effective for TYP-CI may improve visual function for PPCS-CI. Yet, a properly powered randomized clinical trial investigating the effectiveness of OBVAT for PPCS-CI is not available, nor are studies that compare different doses, or provide an understanding of the neural mechanism by which therapeutic interventions may be remediating symptoms and improving vergence function.

Office-Based Vergence and Accommodative Therapy (OBVAT) studied in many prior RCT[45,46,48] has four phases. The first phase will be designed to manage some of the visual/vestibular issues common in PPCS-CI using eye movements associated with head and body movement. In phase 2, techniques will be used to stimulate gross convergence, positive fusional vergence and monocular accommodative therapy using Brock string, Vectograms, Computer Orthoptics and Lens Accommodative Rock, respectively. Once the subject can perform these tasks with ease, s/he will graduate to the third phase which is ramp fusional vergence and monocular accommodative therapy with Vectograms, Aperture Rule, Lifesaver Cards and Lens Accommodative Rock. The final stage will consist of jump fusional vergence and binocular accommodative facility. The goal of the initial phase is to remediate vestibular issues if present. If there are no vestibular issues, then this phase will be omitted.

Home reinforcement will be used to augment subjects' progress in OBVAT. The home

reinforcement software stimulates disparity convergence, and sequences the therapy from ramp to step vergence demands, always providing immediate and accurate feedback to the patient which has been shown to improve vision function.[57] The software also allows the investigator to monitor compliance and performance with the prescribed treatment by logging the number and time of sessions.

Standard-Community Concussion Care (SCC) consists of physical and cognitive rest immediately following the injury for a brief period of time to allow symptoms to abate, followed by a gradual reintroduction of academic and physical activities, restricting activities at high risk for repeat brain injury (such as contact or collision sports) until a graded return to play protocol has been completed without provoking symptoms. For those patients who still have vestibular signs and symptoms persisting beyond 1-month post injury, initiation of vestibular and balance rehabilitation (VBT) physical therapy may occur, as determined by the physician with the goal of improving motion tolerance and balance through exercises that include adaption by retraining the vestibular ocular reflex. While some physical therapists incorporate pencil pushups or Brock string (common treatments for CI) into their VBT protocols, they will be instructed specifically not to include any convergence exercises targeting CI.

This study will incorporate two objective eye movement experiments with corresponding fMRI experiments: VRS3 and VET. The VRS3 and VET will be used to determine the physiological similarities or differences between TYP-CI and PPCS-CI, while the corresponding fMRI experiments will reveal the brain-behavior relationship.

1.4 Compliance Statement

This study will be conducted in full accordance all applicable Children's Hospital of Philadelphia Research Policies and Procedures and all applicable Federal and state laws and regulations including 45 CFR 46. All episodes of noncompliance will be documented.

The investigators will perform the study in accordance with this protocol, will obtain consent and assent, and will report unanticipated problems involving risks to subjects or others in accordance with The Children's Hospital of Philadelphia IRB Policies and Procedures and all federal requirements. Collection, recording, and reporting of data will be accurate and will ensure the privacy, health, and welfare of research subjects during and after the study.

2 STUDY OBJECTIVES

The purpose of the study is to determine the neurophysiology of and treatment for PPCS-CI.

2.1 Primary Objective (or Aim)

The primary objective of this study is to determine the neural mechanistic differences between PPCS-CI and TYP-CI.

2.2 Secondary Objectives (or Aim)

The secondary objectives are to:

- Determine the underlying neural mechanism of OBVAT administered to PPCS-CI patients.
- Compare the effectiveness of standard-community concussion care (SCC) plus 12 vs.16 one-hour OBVAT sessions compared to SCC alone.

3 INVESTIGATIONAL PLAN

3.1 General Schema of Study Design

This study is a randomized controlled trial. Please see Table 1 and Figure 1 for further descriptions of study design.

3.1.1 Screening Phase and Initial Assessment

All patients who present to the Children's Hospital of Philadelphia or Somerset Pediatric Group with a concussion date of injury 1-3 months ago will be screened for enrollment as case subjects using the protocol inclusion and exclusion criteria. Clinicians specializing in concussion management will identify potential subjects under their care in clinics after a standard clinical evaluation that consists of a detailed history and examination. A member of the study team will introduce the study to subjects deemed eligible to participate. Participants will provide verbal consent to screening over the telephone and will complete the majority of the screening process over the phone. Those participants who pass the phone portion of the screening will be asked to complete the vision assessment portion of the screening in person. Eligible subjects will have time to ask questions and consider participation prior to consenting. Parental/guardian permission (informed consent) and, if applicable, child assent, will be obtained prior to any study related procedures being performed.

After consenting to participate, subjects will complete an initial optometric examination, vision assessments, fMRI, and a patient-reported symptom questionnaire. The optometric examination will be completed by an optometrist.

3.1.2 Study Treatment Phase

Case subjects who enroll in the study will be randomized into two groups, one completing SCC+OBVAT and the other completing only SCC in phase 1. A member of the study team will assign subjects to one of the two arms using a random number generator from a computer program. After randomization to a treatment arm, subjects will be provided with instructions on study procedures based on their treatment arm. Subjects in the SCC+OBVAT group (arm 1) will be contacted by a trained and certified vision therapist to schedule and complete their OBVAT sessions. The vision therapist will conduct all OBVAT sessions with participants and will instruct them on the in-home reinforcement. Subjects in both arms will continue to follow-up with the physician treating their concussion as applicable. After the

first 6 weeks and completion of outcome visit 1, subjects in the SCC only group (arm 2) will be contacted by a trained vision therapist to schedule and complete their OBVAT sessions.

3.2 Allocation to Treatment Groups and Blinding

A member of the study team will randomize case subjects who consent to participate into one of the treatment groups at the conclusion of their initial assessment using a random number generator. Physicians will be blinded to the treatment group and will monitor subjects enrolled into the study with standard of care clinical procedures.

3.3 Study Duration, Enrollment and Number of Sites

3.3.1 Duration of Subject Study Participation

The study duration per subject will be up to 11 weeks +/- 3 weeks for arm 1 and 17 weeks +/- 3 weeks for arm 2. With up to 7 days screening, 6 weeks +/- 2 weeks Phase 1, and up to 8 weeks +/- 2 weeks Phase 2 for arm 2 and 2 weeks +/- 1 week for Phase 2 arm 1.

3.3.2 Total Number of Study Sites/Total Number of Subjects Projected

Subject recruitment and data collection will be conducted at The Children's Hospital of Philadelphia and Somerset Pediatric Group. Some subjects may complete their fMRI at the Rutgers University Brain Imaging Center, and the New Jersey Institute of Technology will serve as the data management site.

Recruitment will stop when approximately 150 subjects are enrolled. It is expected that approximately 150 subjects will be enrolled to produce 100 evaluable subjects. We seek to enrolled 50-75 subjects at CHOP and 50-75 subjects at Somerset Pediatric Group. Data from 50 controls (average age of 21 years) have already been collected. Some subjects may not complete the protocols and other datasets may not be analyzed due to motion artifacts within the MRI scanner.

3.4 Study Population

3.4.1 Inclusion Criteria (examples)

3.4.2 Index/Case Subject Inclusion Criteria

1) Males or females age 15 to 25 years.

2) Best-corrected visual acuity of 20/25 in both eyes at distance and near.

3) Willingness to wear glasses or contacts to correct refractive error, if needed.

4) Global stereopsis of 500 sec of arc or better using Randot Stereo Test and local stereopsis of 70 sec of arc or better.

5) Symptomatic PPCS-CI defined as:

    - Exophoria at near more than 4Δ> than at far

- Near point of convergence (NPC) ≥6cm
- Positive fusional vergence (PFV) at 40cm not meeting Sheard's criteria of at least twice the near phoria or a range of ≤ 15Δ
- CI symptom survey score (CISS) of ≥21 for adults and ≥16 for children
- Diagnosis of a concussion from a clinician where symptoms persist for 1 to 3 months post injury

6) Parental/guardian permission (informed consent) and if appropriate, child assent.

3.4.3 Index/Case Subject Exclusion Criteria

1) Previous office or home-based vision therapy, orthoptics, home-based near-target push-ups, or pencil push-ups.

2) Amblyopia or constant strabismus or strabismus surgery.

3) High refractive error: Myopia ≥ 6.0D sphere; Hyperopia ≥ 5.0D sphere; Astigmatism ≥4.0D; Anisometropia >1.5D difference between eyes; prior refractive surgery.

4) Manifest or latent nystagmus evident clinically.

5) Systemic diseases that affect accommodation, vergence or ocular motility (i.e. multiple sclerosis, Graves' thyroid disease, myasthenia gravis, diabetes, chemotherapy, or Parkinson's disease).

6) Any medication known to affect accommodation or vergence.

7) Near point of accommodation >20cm

8) Vertical heterophoria greater than 1Δ.

9) Non-removable metal in the body.

10) Subjects who are pregnant, planning on becoming pregnant during the study duration, or breastfeeding.

11) Subjects who are metal workers.

12) Parents/guardians or subjects who, in the opinion of the Investigator, may be non-compliant with study schedules or procedures.

13) Non-English speakers.

Subjects that do not meet all of the enrollment criteria may not be enrolled. Any violations of these criteria must be reported in accordance with IRB Policies and Procedures.

4 STUDY PROCEDURES

4.1 Screening Visit

- Screening consent
- Screening interview
- Medical record review (cases only)
- Vision exam
- Informed Consent/Assent/HIPAA authorization

4.2 Study Treatment Phase

All subjects who are deemed eligible and interested in participating in the study after the screening will complete a written or electronic informed consent at their screening visit, before any study procedures take place. Subjects will then complete an initial assessment prior to the start of the treatment phase. The initial assessment may take place on the same day as the screening visit after informed consent has taken place.

For arm 1, the study treatment phase will consist of 12 one-hour OBVAT sessions bi-weekly followed by Outcome Visit 1. After completion of Outcome Visit 1, arm 1 subjects will complete 4 additional one-hour OBVAT sessions bi-weekly followed by Outcome Visit 2.

For arm 2, subjects will complete 6 weeks of SCC followed by Outcome Visit 1. After completion of Outcome Visit 1, arm 2 subjects will complete 16 one-hour sessions of OBVAT bi-weekly followed by Outcome Visit 2.

4.2.1 Initial Assessment

- Optometric Exam
- CISS-Con and VisQuaL-T
- VRS3 objective eye movement test
- VET objective eye movement test

4.2.2 fMRI

- Pregnancy test
- MRI anatomical scan and fMRI

4.2.3 Between Initial Assessment and Outcome Visit 1 (Arm 1)

- 12 one-hour bi-weekly OBVAT sessions
- Completion of at-home reinforcement (10-15 min sessions 3 times per week on the days not performing OBVAT while someone is enrolled in OBVAT)

4.2.4 Between Initial Assessment and Outcome Visit 1 (Arm 2)

- Standard-Community Concussion Care

4.2.5 Outcome Visit 1

- Clinical Optometric and medical record Exam
- CISS-Con and VisQuaL-T
- VRS3 objective eye movement test
- VET objective eye movement test
- MRI anatomical scan and fMRI

4.2.6 Between Outcome Visits (Arm 1)

- 4 one-hour bi-weekly OBVAT sessions
- Completion of at-home reinforcement (10-15 min sessions 3 times per week on the days not performing OBVAT while someone is enrolled in OBVAT)

4.2.7 Between Outcome Visits (Arm 2)

- 16 one-hour bi-weekly OBVAT sessions
- Completion of at-home reinforcement (10-15 min sessions 3 times per week on the days not performing OBVAT while someone is enrolled in OBVAT)

4.2.8 Outcome Visit 2

- Clinical Optometric and medical record Exam
- CISS-Con and VisQuaL-T
- VRS3 objective eye movement test
- VET objective eye movement tests
- fMRI*

*for arm 2 subjects only

4.3 Subject Completion/Withdrawal

Subjects may withdraw from the study at any time without prejudice to their care. They may also be discontinued from the study at the discretion of the Investigator for lack of adherence to study treatment or visit schedules. The Investigator or the Sponsor may also withdraw subjects who violate the study plan, or to protect the subject for reasons of safety or for administrative reasons. It will be documented whether or not each subject completes the clinical study. If the Investigator becomes aware of any serious, related adverse events after the subject completes or withdraws from the study, they will be recorded in the source documents and on the CRF.

4.3.1 Early Termination Study Visit

Subjects will be withdrawn if their eligibility status changes such that it is no longer safe for them to complete the fMRI. If subjects choose to withdraw from the study, all of their longitudinal data will be excluded from the final data analysis. If initial baseline assessments are complete a group level comparison will be conducted with this current PPCS-CI data and the already collected TYP-CI and control data.

5 STUDY EVALUATIONS AND MEASUREMENTS

5.1 Screening and Monitoring Evaluations and Measurements

5.1.1 Medical Record Review (case subjects)

- Medical record number
- Name
- Email address
- Phone number
- Date of birth
- Age
- Sex
- Zip code
- Race
- Ethnicity
- Vitals (height, weight, BP and HR if obtained clinically)
- Date of visits
- Concussion history
- Medical history
- Family medical history (family members not directly identified)
- Medications
- Symptoms (pre-injury, initial, and current)
- Injury details
- Activity history
- Clinical examination
- Other concussion assessments administered clinically

- Clinical notes (i.e. return to school/exercise/sports, compliance with therapies, treatment plan)

- Physical therapy notes (if attending therapy at one of the sites or notes scanned into the medical record)

5.1.2 Imaging

Subjects will undergo fMRI imaging exams in the Dept. of Radiology at CHOP or at the Rutgers University Brain Imaging Center. As previously noted, brain imaging data will be obtained from the participants in arm 1 twice and those in arm 2 three times.

5.1.2.1 Functional Magnetic Resonance Imaging Protocol

Magnetic resonance imaging will be performed using an MRI scanner in the Department of Radiology at Children's Hospital of Philadelphia and at the Rutgers University Brain Imaging Center. All MRI systems to be used are FDA-approved systems and are capable of clinical use. See attached FDA approval for Siemens Prisma FDA approval number K202014. Some pulse sequences are not FDA-approved. These non FDA-approved MR sequences were developed and provided by Siemens or collaborators developing sequences for use on Siemens MR systems. These sequences are monitored and controlled by the MR safety systems in the same way as product sequences. These non-FDA approved sequences are widely used for research purposes only, however, not for clinical purposes. The documentation accompanying a Siemens non-FDA approved Works in Progress (WIP) sequence indicates: *"The sequences contained in this software package do not exceed the FDA safety performance parameter guidelines for MRI exams. Specifically, there is no change to patient risk as compared to routine operation of the MAGNETOM with regard to: static magnetic field; the time rate of change of the gradient magnetic fields; the rate at which RF power is deposited into the body (SAR); or the acoustic noise created by the MAGNETOM."* Broadly speaking, non-FDA approved sequences have scientifically useful options and capabilities for adjusting the pulse sequence which have not been tested for FDA approval. However, there is no difference in safety between non-FDA sequences and FDA approved sequences. This is because the MRI scanner system (Prisma) is FDA approved and its safety software enforces the same hardware safety limits and rules for all pulse sequences, regardless of FDA approval status. All documentation on the pulse sequences is attached.

All brain MR images will be read by board certified pediatric neuroradiologists, and all clinically significant incidental findings will be communicated to the participants by the study investigator.

The imaging experiment will have an 8-minute magnetization-prepared rapid acquisition gradient-echo (MPRAGE), 15 minutes of eye movement experiments, and 10 minutes of resting state data collected. Participants will be given breaks between scans and eye movement experiments. An Eyelink-1000 system will be used to record participants' eye movements to ensure that the participant performs the requested visual tasks.

The eye movement experiments will use a set of eccentric squares that stimulate 4° and 6° symmetrical disparity vergence step responses analogous to the behavioral experiment. The

stimulus target extends 2° by 2°. The timing protocol begins with a "rest" block of sustained fixation for 21 seconds. Eight vergence step stimuli are then presented between 2 to 5 seconds per eye movement using the following order: 4° convergence (Con), 4° Con, 6° divergence (Div), 4° Con, 4° Con, 6° Div, 4° Con, and 4° Div. The vergence eye movement task block is 19 seconds in duration. The stimuli will begin with sustained fixation and alternate with vergence response blocks for 10.5 cycles. There will be 210 volumes (full brain images) collected or 420 sec of image acquisition. The protocol will be repeated for saccades of 5° or 10° into the left or right visual field.

5.1.2.2 Pregnancy Testing

A urine pregnancy test will be performed for female subjects who are physically capable of becoming pregnant.

5.1.3 Other Evaluations, Measures

- ***Clinical Examination:*** A standard optometric clinical exam that includes near point of convergence, positive fusional vergence, dissociated phoria, corrected visual acuity, unilateral cover test, stereopsis, monocular amplitude of accommodation, accommodative facility, vergence facility at near and far, and negative fusion vergence will be performed by a practicing, licensed optometrist.

- ***Patient-Reported Outcome Surveys:***
    - CISS-Con
    - Vision Quality of Life with Time Survey (VisQuaL-T)

- ***VET:*** An ISCAN eye tracker integrated into an Oculus head mounted display DK2 will track and record subjects' eye movement. The VET is an 11 minute protocol comparing vergence steps at the beginning and end of a test when distracting stimuli are presented.

- ***VRS3:*** An ISCAN eye tracker integrated into an Oculus head mounted display DK2 will track and record subjects' eye movement. The VRS3 is a 15 minute protocol of vergence ramps, vergence steps, saccades and smooth pursuit objective eye movements.

  - ***Intervention:*** This study involves a therapeutic intervention testing the effect of OBVAT in adolescent and young adults with PPCS-CI. Subjects will be randomly assigned to either an SCC+OBVAT or SCC only group using a pre-determined block randomization procedure.
    - ***SCC+OBVAT:*** subjects assigned to the SCC+OBVAT arm will attend bi-weekly OBVAT sessions with a trained and certified vision therapist. OBVAT has four phases. The first phase will be designed to manage some of the visual/vestibular issues common in PPCS-CI using eye movements associated with head and body movement. In phase 2, techniques will be used to stimulate gross convergence, positive fusional vergence and monocular accommodative therapy using Brock string, Vectograms, Computer

Orthoptics and Lens Accommodative Rock, respectively. Once the subject can perform these tasks with ease, s/he will graduate to the third phase which is ramp fusional vergence and monocular accommodative therapy with Vectograms, Aperture Rule, Lifesaver Cards and Lens Accommodative Rock. The final stage will consist of jump fusional vergence and binocular accommodative facility. The initial phase is used to remediate vestibular issues if preset but if there are no vestibular issues, then this phase will be omitted.

- ***SCC only***: subjects assigned to the SCC only arm will be instructed to follow care instructions of their treating physician. SCC typically consists of physical and cognitive rest immediately following the injury for a brief period of time to allow symptoms to abate, followed by a gradual re-introduction of academic and physical activities, restricting activities at high risk for repeat brain injury (such as contact or collision sports) until a graded return to play protocol has been completed without provoking symptoms. For those patients who still have vestibular signs and symptoms persisting beyond 1-month post injury, initiation of vestibular and balance rehabilitation physical therapy (VBT) may occur, as determined by the physician with the goal of improving motion tolerance and balance through exercises that include adaption by retraining the vestibular ocular reflex. While some physical therapists incorporate pencil pushups or Brock string (common treatments for CI) into their VBT protocols, they will be instructed specifically not to include any convergence exercises targeting CI.

- ***Home reinforcement:*** Subjects will be instructed on in-home reinforcement while they are completing their OBVAT intervention. The home reinforcement will utilize the HTS software. Subjects will be provided the HTS software in the form of a USB drive or a CD. HTS stimulates disparity convergence and sequences the therapy from ramp to step vergence demands, always providing immediate and accurate feedback to the patient, which has been shown to improve vision function. The HTS software also allows the investigator to monitor compliance and performance with the prescribed treatment by logging the number and time of sessions.

5.2 Efficacy Evaluations

5.2.1 Diagnostic Tests, Scales, Measures, etc.

Subjects will complete an optometric examination, vision assessments, fMRI and symptom report at all assessment visits (except arm 1 will not perform an fMRI at the second outcome). Data obtained from these assessments will be tracked and compared between the start and end of the treatment phase for all case subjects enrolled in the study, regardless of treatment arm.

5.3 Safety Evaluation

This is a minimal risk study. The Principal Investigator will be responsible for monitoring the safety of study subjects and complying with all reporting requirements. The PI and study investigators will monitor data accuracy and identify ways to resolve any problem areas. A trained and certified vision therapist will monitor all subjects during OBVAT.

The effects of an MRI on pregnancy or a fetus are known to be potentially harmful from laboratory animal and from human studies. For this reason, subjects undergoing an MRI procedure should not be pregnant at the time of the study. Therefore, subjects who are menstruating will follow CHOP Radiology policies regarding pregnancy testing for the MRI.

6 STATISTICAL CONSIDERATIONS

6.1 Primary Endpoint

The primary endpoint will be the change in composite clinical parameters of near point of convergence and positive fusional vergence between the initial assessment and the outcome visits.

6.2 Secondary Endpoints

Secondary endpoints will include the rate of symptom remediation in OBVAT+SCC group compared to SCC alone, change in vision function, and fMRI measures over the duration of the intervention.

6.3 Statistical Methods

6.3.1 Baseline Data

Baseline and demographic characteristics will be summarized by standard descriptive summaries (e.g. means and standard deviations for continuous variables such as age and percentages for categorical variables such as gender).

6.3.2 Efficacy Analysis

The primary analysis will be based on an intention to treat approach and will include all subjects randomized at their initial assessment.

The primary efficacy endpoint will be the change in composite clinical parameters of near point of convergence and positive fusional vergence between the initial assessment and the outcome visits. A repeated measures ANOVA will be used to assess the clinical parameters as well as the chi-squared test using predetermined published optometric benchmarks for improvement.

Secondary endpoints will include the rate of symptom remediation in OBVAT+SCC group compared to SCC alone, change in vision function, and fMRI measures between the start and end of the treatment phase.

6.3.3 Safety Analysis

All subjects entered into the study at Visit 1 will be included in the safety analysis. The frequencies of AEs by type, body system, severity and relationship to study drug will be summarized. SAEs (if any) will be described in detail.

AE incidence will be summarized along with the corresponding exact binomial 95% two-sided confidence intervals.

6.4 Sample Size and Power

We expect that approximately 150 subjects will be enrolled to produce 100 subjects who finish the protocol and data does not suffer motion artifacts which can cause data to be

omitted from group level analyses. The sample size calculation was performed using paired *t*-tests with equal variance for TYP-CI patients from both placebo therapy and OBVAT. Our published pilot data had an estimated correlation of -0.3, 0.9, -0.4, and -0.3 for CISS, NPC, PFV, beta weight from oculomotor vermis respectively. This gave a standard deviation of the difference (OBVAT- placebo/sham therapy) to be 19, 2, 19, and 27. Assuming 80% power, Alpha= 0.05, and adjusting for an 80% retention rate and 15% data loss due to motion artifacts during fMRI experiments, results in the number of subjects needed to be 37, 6, 37, 46 respectively. Using the maximum sample size for all conditions and to be satisfied, yields a cohort size of 46 per arm. We rounded the number of participants to 50 PPCS-CI for each arm.

6.5 Interim Analysis

A trained vision therapist will monitor and administer the OBVAT to ensure that all participants complete it safely. The treating provider will determine if it is appropriate to terminate the OBVAT for any subjects that show an extended increase in symptoms.

7 STUDY INTERVENTION

7.1 Description

This study involves a therapeutic intervention testing the effect of OBVAT in adolescents and young adults with a PPCS-CI. Subjects will be randomly assigned to either SCC+OBVAT or SCC only arm using a random number generator. Subjects in the SCC+OBVAT arm will be asked to complete 12 one -hour biweekly OBVAT sessions in Phase 1 and an additional 4 one-hour biweekly OBVAT sessions in Phase 2. Subjects in the SCC only arm will be asked to follow SCC as instructed by their clinician for 6 weeks. Subjects will also be asked to complete at-home reinforcement during their OBVAT intervention.

All subjects will complete an optometric clinical exam, a self-reported symptom survey, fMRI and a suite of vision assessments at their study visits.

7.1.1 Treatment Compliance and Adherence

Subjects must complete all of their OBVAT sessions and assessments to receive full compensation for participation in the study. Outcome visits will not be scheduled until the subject has completed all of their OBVAT sessions, and compensation will be dispensed upon completion of each outcome visit.

8 SAFETY MANAGEMENT

8.1 Clinical Adverse Events

Clinical adverse events (AEs) will be monitored throughout the study.

8.2 Adverse Event Reporting

Since the study procedures are not greater than minimal risk, SAEs are not expected. If any unanticipated problems related to the research involving risks to subjects or others happen during the course of this study (including SAEs) they will be reported to the IRB in accordance with CHOP IRB SOP 408: Unanticipated Problems Involving Risks to Subjects. AEs that do not meet prompt reporting requirements will be summarized in narrative or other format and submitted to the IRB at the time of continuing review (if continuing reviews are required), or will be tracked and documented internally by the study team but not submitted to the IRB (if continuing reviews are not required).

9 STUDY ADMINISTRATION

9.1 Treatment Assignment Methods

9.1.1 Randomization

A member of the study team will randomize case subjects who consent to participate into one of the treatment groups at the conclusion of their initial assessment. Participants will be randomly allocated by a member of the study team using a random number generator. Subjects will be randomized in a 1:1 ratio. Block sizes will be multiples of 4, generated by MATLAB, to reduce the potential size of imbalance across the 2 intervention arms. All participants will be assigned a unique identification number at enrollment, which the study team will use to track subjects' date of enrollment, assigned treatment group, and timeline of their treatment phase.

9.1.2 Blinding

Physicians specializing in concussion will identify subjects and then refer them to trained members of the study team who will handle the consent and randomization processes. Physicians will not know which treatment arm each subject is assigned to and will continue routine clinical care for all subjects. Licensed, practicing optometrists will complete the vision assessments, with a different optometrist completing outcome visit 1 and outcome visit 2 for each participant in order to remain blinded.

9.1.3 Unblinding

Physicians treating subjects will be unblinded at the end of the subjects' participation in the study. The optometrists completing the optometric examinations will be unblinded at the completion of the entire study.

9.2 Data Collection and Management

1. **Confidentiality.** Data will be managed and stored using the research-focused electronic data capture system REDCap, under an agreement with the software's development consortium, led by Vanderbilt University. REDCap supports two secure, web-based applications designed exclusively to support data capture for research studies. REDCap is a PHP web application served by Apache Tomcat over a 128-bit SSL connection using a signed certificate. The application relies on a study-specific data dictionary defined in an iterative self-documenting process that will be conducted by all members of the research team. The data dictionary is the foundation for custom case report form design and validated coding of variables. Authentication of research staff will be performed via LDAP using CHOP's enterprise Active Directory service. The application generates a complete audit trail of user activity, provides reporting, and has an automated export mechanism to common statistical packages (SAS, SPSS, Stata, R/S-Plus). Subjects at each site will be assigned a unique ID code and only the local study team at each individual site will be able to view the link between the unique ID and direct patient identifiers. A separate master list will be maintained as a password-protected file on a secure drive at each site and will contain the subject ID number, name, MRN, date of birth and dates of service.

During testing, data may be collected on coded paper forms that include a limited data set, including relevant dates (i.e. date of birth, date of injury, and dates of testing) as well as email and phone number. After the completion of testing, these data will be transferred into the REDCap and the paper forms will either be stored securely in a locked research office or scanned onto the secure research server at each site and destroyed. In addition, each of the objective assessments results in an electronic test report from the specific device utilized. Results from these tests will be stored on the secure storage network at each site and may be input by the study team into the same REDCap database utilized for standard clinical data.

In order to keep protected health information (PHI) from disclosure, all data collected as part of eye movement recordings or MRI will be coded and identified only by a code label assigned to the participant upon entry into the study. When a need for further medical evaluation or treatment is identified during the research, participants will be appropriately referred and upon obtaining consent, the information made available to the health-care provider.

2. **Security**. The REDCap MySQL database is replicated in real time to a completely redundant instance of MySQL. The redundant instance is available for restoration of the primary database or for manual failover in the case of primary database failure. Time-stamped backup files are made from the replicated database daily by CHOP Research Information Systems using automated backup routines. Backup files are encrypted and transferred to a secure file server accessible only to designated personnel. A rolling seven-day window of backup files is maintained in an immediately available online state, with a larger window maintained in a compressed file archive available at a reduced speed of access. Daily destructive database backup files are stored on the database server and are deleted only after successful backup of the entire database to file. In the event of data error, loss or corruption, Research personnel will work with CHOP Research Information Systems to determine the most appropriate recovery strategy. Data and backups are stored in the CHOP Research Information Systems Storage Area Network (SAN). Access to the SAN directories where data are stored will be limited to Research Information Systems personnel, with authentication performed using CHOP's enterprise Active Directory service. Access to the REDCap database will be limited to members involved in the study, and each site will only have access to their own site level data. The master sheet linking PHI with the unique subject ID will be maintained securely at each site so only members of the study team can access.

   The data from the objective eye movement recordings will be stored locally on the computer hard drive that is encrypted and password protected. Data will be automatically saved to an external hard drive that is also encrypted, and password protected, and automatically saves unidentifiable data to the HIPPA compliant enabled via Sookasa inside DropBox shared folders. Everything placed in that

folder is encrypted using 256-bit Advanced Encryption Standard (AES). The Sookasa folder inside the Dropbox sync folder allows the transfer of data to be automatic and encrypted. To view a file, it is downloaded and decrypted locally using the Sookasa App. Sookasa allows users to share files and subfolders, set a password, manage permissions in real time, and set expirations on encryption keys. With Sookasa, a second layer of encryption is created that is HIPAA compliant. Even if there is a compromise within Dropbox itself, the data would not be visible to anyone other than the research team with the appropriate password and security access. Sookasa does not store files on its servers but manages the encryption key distribution, access control, and audit trail collection to ensure a HIPPA compliant system.

All MRI images will be anonymized and stored on a password protected CHOP research servers and/or password protected PCs. MRI images may be shared with NJIT via mailed CDs or the secure sharing system Ambra.

The data collected on paper forms during testing will be secured in a locked research office.

3. **Anonymization, de-identification or destruction**. All PHI collected from this study will be retained until the study is completed, including full data acquisition, analysis, and publication. PHI will only be retained after completion of the study if subjects have indicated a willingness to be contacted for future studies or to future use of their data. We will maintain a separate master sheet with PHI for subjects who agree to future contact or future use of their data. Coded data will be maintained separately and will only be linked to PHI through the subject ID. Subjects who do not consent to future contact or the future use of their data will have their data destroyed 6 years following publication.

9.3 Confidentiality

All data and records generated during this study will be kept confidential in accordance with Institutional policies and HIPAA on subject privacy and that the Investigator and other site personnel will not use such data and records for any purpose other than conducting the study.

No identifiable data will be used for future study without first obtaining IRB approval or determination of exemption. The investigator will obtain a data use agreement between the provider (the PI) of the data and any recipient researchers (including others at participating sites) before sharing a limited dataset (PHI limited to dates and zip codes).

9.4 Regulatory and Ethical Considerations

9.4.1 Data and Safety Monitoring Plan

The CHOP PI will monitor and review the study progress, subject safety, and the accuracy and security of the emerging data and will report any adverse events in accordance with IRB

policies. The PI and members of the study team will review the data once 5 case subjects have completed the study to assess for any potential harm.

9.4.2 Risk Assessment

Risks of this study are not greater than minimal. There are no known medical risks to subjects associated with OBVAT. However, subjects may experience mild discomfort or exacerbation of concussion symptoms which should resolved within a few hours.

There are no known medical risks to subjects associated with functional magnetic resonance imaging (fMRI). However, subjects may experience mild discomfort or feelings of claustrophobia. Each subject will be provided with a squeeze ball alarm as well as voice intercom to indicate that they would like to stop the fMRI scan. Ear plugs will be provided to reduce the noise of the fMRI scan. There is a risk that the vision tasks completed in the fMRI may cause visual discomfort due to eye strain or fatigue or may temporarily provoke or worsen concussion symptoms. Subjects will be given breaks between tasks. This research MRI will include some non-FDA-approved sequences, but all sequences will be administered on an FDA-approved MR scanner. Since the FDA-approved scanner performs safety checks on both FDA- and non-FDA-approved sequences, there should be no risk differential between the two types of sequences.

There is a risk that the optometric exam may temporarily provoke or worsen concussion symptoms; however, these tests will not make the brain injury more severe. Subjects will be given the opportunity to curtail tasks if they provoke symptoms beyond a tolerable threshold.

There is a risk that the objective eye movement recordings may make subjects feel eye fatigue or eye strain. In terms of infrared light projected toward the eye, the manufacturer, ISCAN, has provided testing that shows this system is typically at 20% and may be 30% of the safety limited defined by the ANSI Z136 safety specification limit of 10 mW/cm^2. The percentage can vary depending on how the head mounted display is situated on the subject's head. Subjects have control over when the stimulus will be given and can pause at any time if they need a break. They will be comfortably seated in a chair with a head rest. If they feel any symptoms, they can also verbally tell the research assistant to stop the experiment.

There is also a risk that the questionnaire may make subjects feel uncomfortable, but subjects will not have to answer any questions that they would prefer not to.

Safeguards in place to protect the rights and welfare of children enrolled in the study include parental consent and child assent, routine follow-up visits, and management by physicians with extensive experience treating concussion.

9.4.3 Potential Benefits of Trial Participation

There is no direct benefit to subjects from participating in the fMRI scan or in the eye movement recordings. Subjects may derive some benefit in the form of a reduction in

symptoms from participating in the OBVAT intervention as studies have shown that patients who participated in OBVAT report a decrease in symptoms and improved clinical measures. For society, this study may improve understanding about the effect of OBVAT on recovery after concussion. If OBVAT helps recovery, this will have important implications for the treatment of concussion and may help improve care for concussion patients.

9.4.4 Risk-Benefit Assessment

While there is minimal risk to subjects due to potential exacerbation of symptoms while completing OBVAT sessions, this risk is minimized by careful monitoring of all subjects during their sessions and allowing early termination for any suffering a sharp or prolonged increase in symptoms. The potential risk of accidentally disclosing PHI is minimized through the techniques outlined in the "Data Collection and Management" section. We believe the potential benefits to future PPCS-CI patients through improved treatment and outcomes outweigh these minimal risks.

9.5 Recruitment Strategy

Physicians specializing in concussion will identify eligible case subjects in their clinics. Potentially eligible subjects may be identified in these care settings and recruited from there by members of the study team at each site. If eligible subjects are identified in these care settings and there is no member of the study team available to consent them, the study team may follow-up with the families by phone to provide an overview of the study. The study team will coordinate for potentially eligible participants who express interest in the study to receive a telephone call from a member of the study team to provide an overview of the study and, if interested, begin the screening process.

Control subjects were recruited from participating institutions' student population as well as the surrounding local area via recruitment flyers and word of mouth.

The study team will screen potential subjects against the study's inclusion and exclusion criteria. If eligible, a member of the study team will introduce the study, clearly outlining the risks and benefits of participation. Sufficient time will be provided for subjects and families to ask questions and consider participation. Informed consent and, if applicable, child assent, will be obtained prior to any study related procedures being performed.

9.6 Informed Consent/Assent and HIPAA Authorization

A member of the study team and/or the PI will obtain consent and assent, if applicable, from all eligible subjects that agree to participate in the research. Eligible subjects will be consented in one of two ways: using a paper consent form or electronically via REDCap. For all eligible subjects, the consent form will outline the procedures of the study and indicate the risks and benefits of participation. Subjects will be informed that their participation in the study is voluntary and will not impact the quality of care they receive for their concussion. In addition, they will be given sufficient time to ask questions and consider participation. All subjects will have the option to receive a copy of the consent

form, which includes a description of the research and contact information for the study team. Subjects that turn 18 during the course of the study will be re-consented. To ensure that consent and assent are documented in the same location, assent will also be documented as part of the same electronic form if electronic consent is being obtained. The electronic consent form will include a checkbox for consent by subjects who are at least 18 years of age or a parent/legal guardian of subjects under 18. A separate checkbox for assent will be included for subjects between the ages of 15 and 17.

9.6.1 Screening

The study team will obtain verbal consent for screening and HIPAA authorization via telephone from potentially eligible participants who express interest in the study. Therefore, a waiver of documentation of consent and alteration of HIPAA authorization to screen verbally is requested. Sufficient time will be provided for subjects and families to ask questions and consider participation. Subjects will be provided with a hard or electronic copy of the consent to screening form, which includes a description of the data collected during screening and contact information for the study team. The study team will screen potential subjects against the study's inclusion and exclusion criteria. All screening questions that can be asked and answered via telephone will be done so. If potentially eligible participant pass all screening questions asked over the telephone, a final in-person screening appointment will be scheduled to complete a screening vision assessment. If eligible, a member of the study team will introduce the study, clearly outlining the risks and benefits of participation.

9.6.2 Main Study

After a positive screen, the consent process will take place in person in a private area after a member of the study team has explained the research, clearly outlined the risks and benefits of participation, and provided the subjects with sufficient time to ask the study team member questions and consider participation. Subjects will provide consent and/or assent via an electronic signature at the end of an electronic consent form explaining the details and purpose of the study or via a hand-written signature at the end of a paper consent form. The electronic consent form will be administered in REDCap and will include a written signature for consent by subjects who are at least 18 years of age or a parent/legal guardian of subjects under 18. A separate written signature for assent will be included for subjects between the ages of 15 and 17. Subjects completing consent via REDCap will have the option to receive a copy of the signed consent and HIPAA authorization form via email while subjects completing a paper consent form will receive a paper copy of the consent form for their records.

9.6.3 Consent/HIPAA Authorization Plan for Subjects Who Reach Age of Majority

Subjects that turn 18 during the course of the study will be re-consented in either the electronic or paper format.

9.6.4 Waiver of HIPAA Authorization

We believe this study qualifies for an alteration of HIPAA authorization as the use or disclosure of protected health information involves no more than minimal risk to the privacy of individuals. This is due to the fact that there is an adequate plan to protect identifiers and

to destroy identifiers at the earliest opportunity as noted in the section entitled "Data Collection and Management." There is also a written assurance that protected health information will not be reused or disclosed to any other person or entity, except as required by law, for authorized oversight of the research project, or for other research for which the use or disclosure of protected health information would be permitted by this subpart as noted in the section entitled "Confidentiality." Alteration of HIPAA authorization is necessary to obtain verbal HIPAA authorization for the phone screen. The phone screener, and thus the screening portion for this study, cannot practically be conducted without this alteration of HIPAA since PHI will be collected without written consent over the phone. An appropriate plan is in place to protect identifying information from disclosure as well as a plan to destroy identifiers. Additionally, the research cannot practicably be carried out without the alteration because PHI is necessary to identify our target population and review medical records; therefore, this research could not be practicably carried out without access to this information.

9.7 Payment to Subjects/Families

9.7.1 Payments to subject for time, effort and inconvenience (i.e. compensation)

Subjects will be compensated for participation in the study because it requires a significant investment of time and effort. Subjects will receive up to a total of $400 as compensation in the form of a ClinCard. Subjects will receive $100 for completing the initial assessment, $100 for completing outcome visit 1, and $200 for completing outcome visit 2.

10 PUBLICATION

We plan on having the results of this study presented at national or international meetings, published in peer-reviewed academic journals, and potentially included in future grant applications. We will not disclose private health information in any presentation or publication about this study.

11 REFERENCES

1. Younger, D. S. Sports-Related Concussion in School-Age Children. *World J. Neurosci.* **8**, 10–31 (2018).
2. Kelly, J. P. *et al.* Concussion in Sports. *JAMA* **266**, 2867 (1991).
3. Gottschalk, A. W. & Andrish, J. T. Epidemiology of Sports Injury in Pediatric Athletes. *Sports Med. Arthrosc.* **19**, 2–6 (2011).
4. BlueCross BlueShield. The Steep Rise in Concussion Diagnoses in the U.S. *Heal. Am. Rep.* (2016).
5. Cnossen, M. C. *et al.* Prediction of Persistent Post-Concussion Symptoms Following Mild Traumatic Brain Injury. *J. Neurotrauma* neu.2017.5486 (2018). doi:10.1089/neu.2017.5486
6. Boutis, K. *et al.* The Diagnosis of Concussion in Pediatric Emergency Departments: A Prospective Multicenter Study. *J. Emerg. Med.* **54**, 757–765 (2018).

7. Master, C. L., Gioia, G. A., Leddy, J. J. & Grady, M. F. Importance of 'return-to-learn' in pediatric and adolescent concussion. *Pediatr. Ann.* **41**, 1–6 (2012).
8. Dematteo, C. *et al.* A balanced protocol for return to school for children and youth following concussive injury. *Clin. Pediatr. (Phila).* **54**, 783–792 (2015).
9. Hall, E. E. *et al.* Concussion management in collegiate student-athletes: Return-to-academics recommendations. *Clin. J. Sport Med.* **25**, 291–296 (2015).
10. Conrick, K. M. G. *et al.* Community-Engaged Approach to the Development and Implementation of a Student-Centered Return to Learn Care Plan After Concussion. *J. Sch. Health* **90**, 842–848 (2020).
11. Swanson, M. W. *et al.* Academic Difficulty and Vision Symptoms in Children with Concussion. *Optom. Vis. Sci.* **94**, 60–67 (2017).
12. Johansson, J., Nygren de Boussard, C., Öqvist Seimyr, G. & Pansell, T. The effect of spectacle treatment in patients with mild traumatic brain injury: a pilot study. *Clin. Exp. Optom.* **100**, 234–242 (2017).
13. Donker-Cools, B. H. P. M., Schouten, M. J. E., Wind, H. & Frings-Dresen, M. H. W. Return to work following acquired brain injury: the views of patients and employers. *Disabil. Rehabil.* **40**, 185–191 (2018).
14. Graff, H. J., Deleu, N. W., Christiansen, P. & Rytter, H. M. Facilitators of and barriers to return to work after mild traumatic brain injury: A thematic analysis. *Neuropsychol. Rehabil.* (2020). doi:10.1080/09602011.2020.1778489
15. Cancelliere, C. *et al.* Systematic review of return to work after mild traumatic brain injury: Results of the international collaboration on mild traumatic brain injury prognosis. *Archives of Physical Medicine and Rehabilitation* **95**, (2014).
16. Cochrane, G. D. *et al.* Visuo-oculomotor Function and Reaction Times in Athletes with and without Concussion. *Optom. Vis. Sci.* 1 (2019). doi:10.1097/OPX.0000000000001364
17. Craton, N. *et al.* COACH CV: The Seven Clinical Phenotypes of Concussion. *Brain Sci.* **7**, 119 (2017).
18. Ellis, M. J., Leddy, J. J. & Willer, B. Physiological, vestibulo-ocular and cervicogenic post-concussion disorders: An evidence-based classification system with directions for treatment. *Brain Inj.* **29**, 238–248 (2015).
19. Ellis, M. J., Leddy, J. & Willer, B. Multi-disciplinary management of athletes with post-concussion syndrome: An evolving pathophysiological approach. *Front. Neurol.* **7**, (2016).
20. Lumba-Brown, A. *et al.* Representation of concussion subtypes in common postconcussion symptom-rating scales. *Concussion* **4**, (2019).
21. Collins, M. W., Kontos, A. P., Reynolds, E., Murawski, C. D. & Fu, F. H. A comprehensive, targeted approach to the clinical care of athletes following sport-related concussion. *Knee Surgery, Sports Traumatology, Arthroscopy* **22**, 235–246 (2014).
22. Berthold-Lindstedt, M., Ygge, J. & Borg, K. Visual dysfunction is underestimated in patients with acquired brain injury. *J. Rehabil. Med.* **49**, 327–332 (2017).
23. Kerr, Z. Y. *et al.* Factors associated with post-concussion syndrome in high school student-athletes. *J. Sci. Med. Sport* **21**, 447–452 (2018).
24. Starkey, N. J. *et al.* Post-concussive symptoms after a mild traumatic brain injury during childhood and adolescence. *Brain Inj.* **32**, 617–626 (2018).

25. Master, C. L. *et al.* Vision Diagnoses Are Common after Concussion in Adolescents. *Clin. Pediatr. (Phila).* **55**, 260–267 (2016).
26. Brahm, K. D. *et al.* Visual Impairment and Dysfunction in Combat-Injured Servicemembers With Traumatic Brain Injury. *Optom. Vis. Sci.* **86**, 817–825 (2009).
27. Alvarez, T. L. *et al.* Concurrent vision dysfunctions in convergence insufficiency with traumatic brain injury. *Optom. Vis. Sci.* **89**, 1740–1751 (2012).
28. Szymanowicz, D. *et al.* Vergence in mild traumatic brain injury: a pilot study. *J. Rehabil. Res. Dev.* **49**, 1083–100 (2012).
29. Lovell, M. R. *et al.* Measurement of symptoms following sports-related concussion: Reliability and normative data for the post-concussion scale. *Appl. Neuropsychol.* **13**, 166–174 (2006).
30. Letourneau, J. & Ducic, S. Prevalence of Convergence Insufficiency among Elementary School Children. *Can J Optom* **50**, 194–97 (1988).
31. Rouse, M. *et al.* Frequency of Convergence Insufficiency Among Fifth and Sixth Graders. *Optom. Vis. Sci.* **76**, 643–649 (1999).
32. Hussaindeen, J. R. *et al.* Prevalence of non-strabismic anomalies of binocular vision in Tamil Nadu: report 2 of BAND study. *Clin. Exp. Optom.* **100**, 642–648 (2017).
33. Wajuihian, S. O. & Hansraj, R. Vergence anomalies in a sample of high school students in South Africa. *J. Optom.* **9**, 246–257 (2016).
34. Davis, A. L. *et al.* Convergence Insufficiency, Accommodative Insufficiency, Visual Symptoms, and Astigmatism in Tohono O'odham Students. *J. Ophthalmol.* **2016**, 1–7 (2016).
35. Ovenseri-Ogbomo, G. O. & Eguegu, O. P. Vergence findings and horizontal vergence dysfunction among first year university students in Benin City, Nigeria. *J. Optom.* **9**, 258–263 (2016).
36. García-Muñoz, Á., Carbonell-Bonete, S., Cantó-Cerdán, M. & Cacho-Martínez, P. Accommodative and binocular dysfunctions: prevalence in a randomised sample of university students. *Clin. Exp. Optom.* **99**, 313–321 (2016).
37. Capó-Aponte, J. E., Beltran, T. A., Walsh, D. V, Cole, W. R. & Dumayas, J. Y. Validation of Visual Objective Biomarkers for Acute Concussion. *Mil. Med.* **183**, 9–17 (2018).
38. Raghuram, A. *et al.* Postconcussion: Receded Near Point of Convergence is not Diagnostic of Convergence Insufficiency. *Am. J. Ophthalmol.* **206**, 235–244 (2019).
39. Scheiman, M., Grady, M. F. & Jenewein, E. Frequency of Oculomotor Vision Disorders in Adolescents 11 to 17 Years of Age with Concussion, 4 to 12 Weeks Post Injury. *Vision Res.*
40. Gallaway, M., Scheiman, M. & Mitchell, G. L. Vision Therapy for Post-Concussion Vision Disorders. *Optom. Vis. Sci.* **94**, 68–73 (2017).
41. Master, C. L. *et al.* Vision and Vestibular System Dysfunction Predicts Prolonged Concussion Recovery in Children. *Clin. J. Sport Med.* **28**, 1 (2017).
42. Suleiman, A. *et al.* Correlation between Ocular and Vestibular Abnormalities and Convergence Insufficiency in Post-Concussion Syndrome. *Neuro-Ophthalmology* **44**, 157–167 (2020).
43. Scheiman, M. & Wick, B. *Clinical management of binocular vision: Heterophoric, accommodative, and eye movement disorders: Fifth edition*. *Clinical Management of Binocular Vision: Heterophoric, Accommodative, and Eye Movement Disorders:*

Fifth Edition (Wolters Kluwer/Lippincott Williams & Wilkins, 2020).
44. Master, C. L. *et al.* Vision Diagnoses Are Common After Concussion in Adolescents. *Clin. Pediatr. (Phila).* **55**, 260–267 (2016).
45. Alvarez, T. L. *et al.* The Convergence Insufficiency Neuro-mechanism in Adult Population Study (CINAPS) Randomized Clinical Trial: Design, Methods, and Clinical Data. *Ophthalmic Epidemiol.* **27**, 52–72 (2020).
46. Scheiman, M. *et al.* The convergence insufficiency treatment trial: Design, methods, and baseline data. *Ophthalmic Epidemiol.* **15**, 24–36 (2008).
47. Convergence Insufficency Investigator Group. A Randomized Clinical Trial of Treatment for Symptomatic Convergence Insufficiency in Children (Citt-Art). *Optom Vis Sci* **XX**, (2019).
48. CITT-ART Investigator Group *et al.* Convergence Insufficiency Treatment Trial - Attention and Reading Trial (CITT-ART): Design and Methods. *Vis. Dev. Rehabil.* **1**, 214–228 (2015).
49. Scheiman, M., Gwiazda, J. & Li, T. Non-surgical interventions for convergence insufficiency. *Cochrane database Syst. Rev.* CD006768 (2011). doi:10.1002/14651858.CD006768.pub2
50. Convergence Insufficiency Treatment Trial Study Group, C. I. T. T. I. *et al.* Randomized clinical trial of treatments for symptomatic convergence insufficiency in children. *Arch. Ophthalmol.* **126**, 1336–1349 (2008).
51. Scheiman, M. *et al.* A randomized clinical trial of treatments for convergence insufficiency in children. *Arch. Ophthalmol.* **123**, 14–24 (2005).
52. CITT-ART Investigator Group. Treatment of Symptomatic Convergence Insufficiency in Children Enrolled in the Convergence Insufficiency Treatment Trial-Attention & Reading Trial: A Randomized Clinical Trial. *Optom. Vis. Sci.* **96**, 825–835 (2019).
53. Scheiman, M. *et al.* A Randomized Clinical Trial of Vision Therapy/Orthoptics versus Pencil Pushups for the Treatment of Convergence Insufficiency in Young Adults. *Optom. Vis. Sci.* **82**, E583–E595 (2005).
54. Alvarez, TL, Scheiman, M, Santos, EM, Yaramothu, C, d'Antonio-Bertagnolli, J.-V. Convergence Insufficiency Neuro-mechanism in Adult Population Study Randomized Clinical Trial: Clinical Outcome Results. *Optom. Vis. Sci.* (2020).
55. Rollett, P. & Morandi, G. *Effect of Vision Therapy on Measures of Oculomotor Function in Patients Presenting with Post-Concussion Syndrome*. *Canadian Journal of Optometry* **81**, (2019).
56. Scheiman, M., Talasan, H., Mitchell, G. & Alvarez, T. L. Objective Assessment of Vergence after Treatment of Concussion-Related CI: A Pilot Study. *Optom. Vis. Sci.* **94**, 74–88 (2017).
57. Cooper, J. & Feldman, J. Reduction of symptoms in binocular anomalies using computerized home therapy-HTS™. *Optometry* **80**, 481–486 (2009).
